# Leveraging biological and statistical covariates improves the detection power in epigenome-wide association testing

**DOI:** 10.1186/s13059-020-02001-7

**Published:** 2020-04-06

**Authors:** Jinyan Huang, Ling Bai, Bowen Cui, Liang Wu, Liwen Wang, Zhiyin An, Shulin Ruan, Yue Yu, Xianyang Zhang, Jun Chen

**Affiliations:** 1 State Key Laboratory of Medical Genomics, Shanghai Institute of Hematology, National Research Center for Translational Medicine, Rui-Jin Hospital, Shanghai Jiao Tong University School of Medicine, Shanghai Jiao Tong University, 197 Ruijin Er Road, Shanghai, 200025 China; 2 Department of General Surgery, Rui-Jin Hospital, Shanghai Jiao Tong University, 197 Ruijin Er Road, Shanghai, 200025 China; 3 Division of Digital Health Sciences, Mayo Clinic, 200 1st St SW, Rochester, MN 55905 USA; 4 Department of Statistics, Texas A&M University, Blocker 449D, College Station, TX 77843 USA; 5 Division of Biomedical Statistics and Informatics, Department of Health Sciences Research and Center for Individualized Medicine, Mayo Clinic, 200 1st St SW, Rochester, MN 55905 USA

**Keywords:** False discovery rate, EWAS, Multiple hypothesis testing, Covariate

## Abstract

**Background:**

Epigenome-wide association studies (EWAS), which seek the association between epigenetic marks and an outcome or exposure, involve multiple hypothesis testing. False discovery rate (FDR) control has been widely used for multiple testing correction. However, traditional FDR control methods do not use auxiliary covariates, and they could be less powerful if the covariates could inform the likelihood of the null hypothesis. Recently, many covariate-adaptive FDR control methods have been developed, but application of these methods to EWAS data has not yet been explored. It is not clear whether these methods can significantly improve detection power, and if so, which covariates are more relevant for EWAS data.

**Results:**

In this study, we evaluate the performance of five covariate-adaptive FDR control methods with EWAS-related covariates using simulated as well as real EWAS datasets. We develop an omnibus test to assess the informativeness of the covariates. We find that statistical covariates are generally more informative than biological covariates, and the covariates of methylation mean and variance are almost universally informative. In contrast, the informativeness of biological covariates depends on specific datasets. We show that the independent hypothesis weighting (IHW) and covariate adaptive multiple testing (CAMT) method are overall more powerful, especially for sparse signals, and could improve the detection power by a median of 25% and 68% on real datasets, compared to the ST procedure. We further validate the findings in various biological contexts.

**Conclusions:**

Covariate-adaptive FDR control methods with informative covariates can significantly increase the detection power for EWAS. For sparse signals, IHW and CAMT are recommended.

## Background

DNA methylation, as a major form of epigenetic modifications, plays a vital role in various biological processes including cell differentiation [1–3], genomic imprinting [4], gene transcription, and X-chromosome inactivation [5]. The landscape of DNA methylation is not only associated with normal physiological phenomena such as aging [6–8], but also with many human diseases including cancers [8–10], atherosclerosis [11], and Alzheimer’s disease [12, 13]. While the DNA sequences are relatively constant throughout life, DNA methylation is dynamic and modifiable, providing promising therapeutic targets for disease treatment [14–16].

With the advance of high-throughput genomic technologies, DNA methylation can now be interrogated at the genome scale. Although the gold standard for DNA methylation measurement remains bisulfite sequencing [17], DNA methylation arrays, due to its low cost, high reproducibility, and good genome coverage, have been widely used in genome-wide methylation analyses. Illumina’s Infinium Human Methylation 450K BeadChip and EPIC BeadChip, which cover more than 450,000 and 850,000 CpG methylation sites respectively, are two predominant products in the market. The availability of these high-density methylation arrays has fueled epigenome-wide association studies (EWAS), which seek to identify methylation variants associated with an outcome or exposure of interest [18–23]. Analysis of EWAS data typically involves testing all the CpG sites simultaneously, leading to a massive multiple testing problem. Two statistical approaches have been developed to address multiple testing: family-wise error rate (FWER) and false discovery rate (FDR) control. The FWER approach controls the probability of making one or more false discoveries, while the FDR approach controls the expected proportion of false discoveries. Therefore, the FWER approach, such as Bonferroni correction [24], offers a more stringent type I error control but is substantially less powerful than the FDR approach. For EWAS data, the sample size and the expected effect size are usually moderate, making the FDR approach particularly appealing [25–27]. Among existing FDR control procedures, the original Benjamini-Hochberg step-up procedure (BH) [28] and Storey’s *q* value procedure (ST) [29] are the two most popular methods for genome-wide multiple testing. Compared to the BH procedure, the ST procedure considers the proportion of null hypotheses and is more powerful when the signal is dense.

Both BH and ST procedures do not differentiate hypotheses, that is, they assume that each hypothesis is equally likely to be true or false, and their rejection rule is based solely on the *p* values. Therefore, BH and ST procedures may not be optimal when we have additional information about the hypotheses in terms of their null probability (the probability for the null hypothesis to be true) or statistical power. For EWAS data, besides the association *p* values, we have plenty of auxiliary covariates, which could be informative of the null probability or statistical power of the CpG-specific hypotheses. For example, differentially methylated CpG positions (DMPs) have been found to be enriched in specific genomic regions, such as promoters [30, 31], CpG islands [20], shores [32], a specific chromosome [33], and DNase I hypersensitive sites [32, 34]. In addition, some studies found that DMPs tend to change in the same direction, especially in cancer, where genome-wide hypomethylation or hypermethylation has been frequently observed [10]. Other studies found a large portion of the CpGs are subject to large measurement errors, hence low statistical power [35, 36]. Therefore, incorporating such biological or statistical covariates in FDR control could potentially improve the power to detect DMPs.

Recently, there has been a surge in the development of statistical methodologies for FDR control procedures accommodating covariates with the aim to improve the detection power while still maintaining the target FDR level. Such covariate-adaptive FDR control methods include weighted FDR [37], the conditional local FDR (LFDR) [38], FDR regression (FDRreg) [39], independent hypothesis weighting (IHW) [40], adaptive shrinkage (ASH) [41], Boca and Leek’s FDR regression (BL) [42], adaptive *p* value thresholding (AdaPT) [43], and covariate adaptive multiple testing (CAMT) [44]. Although these methods differ in their respective model and input, they share the same idea: by relaxing the rejection criterion for more promising hypotheses based on the covariate information and tightening the criterion for others, substantial power improvement can be achieved without affecting the target FDR level. These covariate-adaptive methods have been demonstrated to be superior to traditional BH/ST procedures by case studies on ChIP-seq, genotype, microbiome, RNA-seq, and scRNA-seq data [45], but application to EWAS data has not yet been attempted. Since these methods rely on the assumption that the hypotheses are independent or weakly dependent, it is not clear whether they are robust to the typical correlation structure observed in EWAS data. Moreover, it is unknown what CpG-related covariates are relevant. Therefore, a rigorous and comprehensive evaluation of the covariate-adaptive FDR methods for EWAS data is critical before recommending them to the field.

In this study, we compared five covariate-adaptive FDR control methods using real data-based simulations and investigated the performance of 14 CpG-related covariates on 61 EWAS datasets. The contribution of the paper is thus threefold: (1) we developed a powerful statistical test for detecting and selecting informative covariates, (2) we identified the most robust and powerful covariate-adaptive FDR control methods for EWAS data, and (3) we recognized the most relevant covariates for EWAS data.

## Results

### Overview of the EWAS datasets and covariates selected for evaluation

In this study, 61 EWAS datasets were collected based on 58 Gene Expression Omnibus (GEO) methylation datasets whose platforms were Infinium Human Methylation 450K BeadChip. The sample size of EWAS datasets ranged from 100 to 689, with an average of 211. Details about the data source and analyzed phenotypes can be found in the additional file (Additional file 1: Table S1). The methylation datasets came from diverse tissue sources, including blood, brain, lung, breast, colorectal, liver, esophagus, and others (Additional file 6: Figure S1). Around half of the datasets were from the blood (30/61). After quality control, we performed surrogate variable analysis (*SmartSVA*) [46] to capture significant sources of methylation variability, such as cellular heterogeneity [47] and batch effects [48]. The constructed surrogate variables were included as covariates in the regression model to account for potential confounding effects. After adjusting for surrogate variables, we observed a significant reduction of the genomic inflation [46] of the association *p* values (Additional file 6: Figure S2). The adjusted *p* values were then to be corrected for multiple testing by various FDR control procedures.

Covariate-adaptive FDR control methods require selection of appropriate covariate(s) for the method to work. To be statistically valid, the covariate should be independent of the *p* values under the null. Meanwhile, in order to increase the detection power, the covariate should be informative of the prior null probability or statistical power of the underlying hypotheses. For EWAS data, we investigated 14 potential covariates, which were assumed to possess the aforementioned properties. These covariates can be classified as statistical covariates (internal) and biological/technical covariates (external). Statistical covariates are related to the statistical properties of the methylation data. We investigated mean of the Beta-value (“mean”), standard deviation of the Beta-value (“sd.b”) or M-value (“sd.m”), median absolute deviation of the Beta-value (“mad”, a robust measure of variance), measure of unimodality of the Beta-value (“dip”), inverse precision parameter of the Beta-value (“precision”) [49], sign of the regression coefficients (“direction”) for binary/continuous phenotype, and intraclass correlation coefficient (ICC) using the Beta-value (“icc.b”), or M-value (“icc.m”) when replicates were available. Biological/technical covariates describe the biological/technical properties of the CpGs and are based on the annotation of the CpG probes. We considered the location in the gene region (“refgene.pos”), relation to the CpG island (“cpg.loc”), chromosome number (“chr”), DNase I hypersensitive site (“dhs”), and Infinium probe type (“probe.type”). Details of the covariates are listed in Table 1.

**Table 1:** Description of the covariates evaluated

Covariate	Definition	Type
mean	Mean Beta-values *(R base::mean)*	Statistical, continuous
sd.b/sd.m^#1^	Standard deviation of Beta-value or M-value *(R stats::sd)*	Statistical, continuous
mad	Median absolute deviation *(R stats::mad)*	Statistical, continuous
dip	Measure of unimodality using dip statistic *(R diptest::dip)*	Statistical, continuous
precision	Inverse precision parameter, *(1/(mean×(1 − mean)/sd*^*2*^ *– 1))*	Statistical, continuous
direction^#2^	Sign of regression coefficients	Statistical, categorical *{positive, negative}*
icc.b/icc.m^#3^	Intraclass correlation coefficient *(R CpGFilter::CpGFilterICC)*	Statistical, continuous
refgene.pos	Position in the gene region	Biological, categorical *{5’UTR, TSS1500, TSS200, 1stExon, Body, 3′-UTR, Non_gene*^*#4*^*}*
cpg.loc	Relation to CpG island	Biological, categorical *{OpenSea, N_Shelf, N_Shore, Island, S_Shore, S_Shelf}*
chr	Chromosome number	Biological, categorical *{chr1, chr2, …, chr22}*
dhs	DNase I hypersensitive site	Biological, categorical *{yes, no}*
probe.type	Infinium probe type	Technical, categorical *{type I, type II}*

#1. The suffix .b and .m stand for Beta-value and M-value, respectively. If not noted, the calculation is based on Beta-value

#2. Used when the phenotype is binary or continuous

#3. Used when there are technical replicates in data

#4. When multiple gene regions are annotated for the CpG, the first one is used. If no annotation is available, we labeled it “Non_gene”

### An omnibus test to assess the informativeness of the covariate for multiple testing adjustment

For a covariate that could be leveraged to increase the power for epigenome-wide multiple testing, it has to be informative of the null probability or the statistical power, either of which will lead to the dependency between the *p* value and the covariate. Such dependency is usually explored by various diagnostic plots such as scatter plot and stratified *p* value histogram [40] (Additional file 6: Figure S3 & 4). Although these diagnostic plots can detect a strong dependency efficiently, they may have limited ability to reveal a more subtle and complex relationship, especially when the signal is sparse. We thus develop a formal statistical test to rigorously assess the dependency between *p* value and the covariate (“Methods”). Basically, the test exploits the assumptions that the signal is usually sparse, and the dependency may be highly nonlinear. This is achieved by testing the association between two categorical variables after dichotomizing the *p* values at the lower end and splitting the covariate into disjoint sets if it is continuous. An omnibus-type test is designed to combine evidence through various categorizations, and permutation is used to assess the statistical significance.

**Fig. 1:**
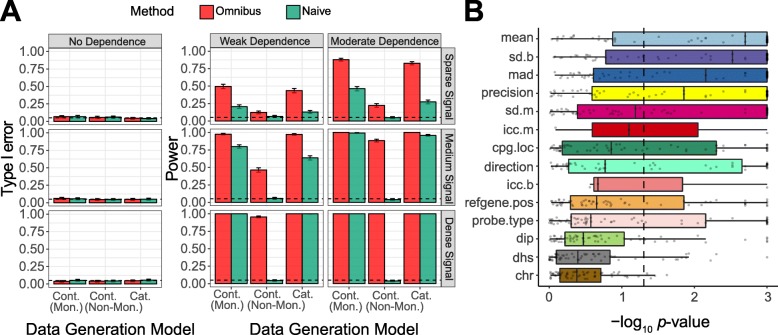
Informativeness assessment by the proposed omnibus test. **a** Performance evaluation of the omnibus test compared with naïve tests. Simulated datasets are used to assess type I error (“No dependence” between the *p* value and the covariate, first column) and power (“Weak dependence” and “Moderate dependence”, second and third column). Varying signal intensities (“Sparse signal,” “Medium signal,” and “Dense signal”, first to third row) as well as different data generation models are studied. “Cont.(Mon.),” “Cont.(Non-Mon),” and “Cat.” represent continuous covariates with a monotonic dependence, continuous covariates with a non-monotonic dependence, and categorical covariates, respectively. Naïve tests refer to Spearman’s rank correlation test and Kruskal-Wallis test for continuous and categorical covariates, respectively. The nominal level for type I error is 0.05 (dashed line). **b** Informativeness of EWAS-relevant covariates as assessed by the omnibus test. Boxplot depicts the distribution of the omnibus test *p* values across datasets. The *x*-axis is on a log scale, and the boxplots are ordered by the median values. The dashed line indicates *p* value 0.05

We first performed simulations to assess the type I error and power of the proposed omnibus test and benchmarked it against the naïve tests—Spearman’s rank test and Kruskal-Wallis test for continuous and categorical covariates, respectively. We generated data with both continuous and categorical covariates and varied the degree of *p* value-covariate dependency and signal density (“Methods”). For a continuous covariate, we investigated both monotonic and non-monotonic dependency. The results are summarized in Fig. 1a. When there was no dependency (the covariate was not informative), both the omnibus and naïve tests controlled the type I error at the nominal level under all settings. When the signal was sparse, the omnibus test was significantly more powerful than the naïve tests. As the signal became denser, the power difference decreased for categorical covariates and continuous covariates under a monotonic relationship. However, when the relationship was non-monotonic, the naïve tests were powerless under all settings. Therefore, the proposed omnibus test was particularly suited for the sparse signal and complex dependency setting, which were expected in many EWAS datasets.

### Evaluation of the informativeness of EWAS-relevant covariates

We next applied the omnibus test to the aforementioned 14 covariates based on the CpG association *p* values from the 61 EWAS datasets. To satisfy the exchangeability assumption of the permutation-based omnibus test, we subsampled the *p* values so that the auto-correlation coefficient was close to 0 between adjacent CpGs. We plotted the distribution of the omnibus test *p* values (log scale) for these 14 covariates to assess their informativeness (Fig. 1b, Additional file 2: Table S2). One clear pattern is that the statistical covariates dominate the biological/technical covariates with overall smaller omnibus *p* values. Interestingly, among the statistical covariates, the mean methylation level (“mean”) was the most informative, followed by the covariates measuring the methylation variance (“sd.b,” “mad,” “precision”). For these covariates, the majority of datasets achieved significant omnibus test *p* values (*p* < 0.05). Additional file 6: Figure S3A&B shows two examples where the small association *p* values were enriched in small and medium Beta-values, respectively. Variance on the methylation Beta-value (“sd.b”) was more informative than that on the M-value (“sd.m”). The direction of the effect (“direction”) had mixed results, with 46.3% (25/54) of the datasets being significant. The DIP statistic (“dip”), on the other hand, was the least informative; only 12 out of 61 datasets had significant *p* values. For the three datasets with replicates, where we could calculate ICC values, we also evaluated the informativeness of the ICC measure (“icc.b” and “icc.m”). One dataset achieved a very low omnibus test *p* value (*p* < 0.001) while the other two were not significant, possibly due to few replicates available and an extremely sparse signal. In contrast, the biological/technical covariates were less “universal”: they were informative only on specific datasets. Overall, the relation to the CpG island (“cpg.loc”) was the most informative, followed by the position in the reference gene (“refgene.pos”) and probe type (“probe.type”). For chromosome location (“chr”) and DNase I hypersensitive site (“dhs”), they were only significant in 4 and 10 datasets, respectively.

A statistically valid covariate needs to be independent of the *p* values under the null [40]. We verified this condition using stratified histograms as suggested by [40]: the distribution of larger *p* values had an approximate uniform distribution across the strata of the covariate. Additional file 6: Figure S3 & S4 gave representative examples for each type of covariate.

### Evaluation of the performance of covariate-adaptive FDR control methods on simulated EWAS datasets

Most of the existing covariate-adaptive FDR methods rely on the independence assumption, i.e., the hypotheses are not correlated. However, for EWAS data, the methylation levels of neighboring CpGs are usually correlated, violating the independence assumption. It is not clear whether these methods can still control FDR at the target level while retaining the high statistical power for EWAS data. We thus use simulation, where we know the ground truth, to study the performance of the methods. To mimic the correlation structure of EWAS data, we used a real EWAS dataset [50] as a template. We drew random samples from the control group to create two groups of equal sample size and added random or correlated differential signals of varying strength and density (“Methods”). We simulated covariates with varying degrees of informativeness and compared the performance of AdaPT, BL, CAMT, FDRreg, IHW, and the traditional BH and ST procedure (Table 2).

**Table 2:** Description of the methods evaluated

	Method description	R package	Ref
BH	The classic procedure. *p* values for *m* hypotheses are ordered from the smallest to the largest. Given a target FDR level *α*, the *i*th hypothesis is rejected if the *p* value is less than the threshold *α*$\frac{i}{m}$.	stats *(p.adjust)*	[28]
ST	The global proportion of null hypotheses is estimated and used to adjust the threshold in the BH procedure. Less conservative than BH when the signal is not sparse.	qvalue *(qvalue)*	[29]
FDRreg	Covariates are allowed to influence the prior probability of null. The rejection rule is based on local false discovery rate (lFDR) under the two-component mixture model.	FDRreg *(FDRreg)*	[39]
IHW	Tests are divided into groups based on the covariate. Each group is associated with a weight, and the weight is used to adjust the threshold in the BH procedure. Only one covariate is allowed.	ihw *(ihw)*	[40]
BL	A regression framework is used to estimate the proportion of null hypotheses conditional on observed covariates. The estimates are used to adjust the threshold in the BH procedure.	swfdr *(lm_pi0)*	[42]
AdaPT	Covariates are allowed to influence both the null probability and the *p* value distribution under the alternative. The rejection rule is based on lFDR and an adaptive approach is implemented to control FDR in finite sample.	adaptMT *(adapt_glm)*	[43]
CAMT	Covariates are allowed to influence both the null probability and the *p* value distribution under the alternative. A new rejection rule is designed to be robust to model mis-specification.	CAMT *(camt.fdr)*	[51]

**Fig. 2:**
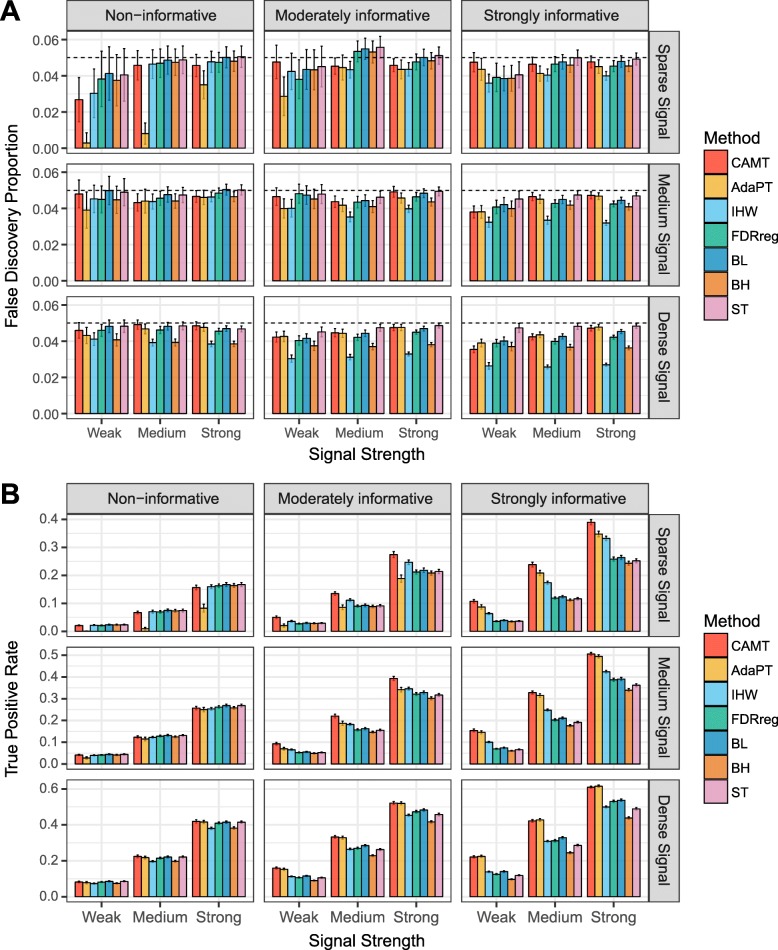
Performance evaluation of covariate-adaptive FDR control methods on simulated data (random signals). Performance is evaluated based on FDR control (average false discovery proportion, panel **a**) and power (average true positive rate, panel **b**). The performance is compared under combinations of different level of covariate informativeness (“Non-informative,” “Moderately informative,” and “Strongly informative,” columns), signal density (“Sparse signal,” “Medium signal,” and “Dense signal” rows), and signal strength (“Weak,” “Medium,” “Strong”). Error bar indicates the standard error across 100 repetitions, and the dashed line denotes the target FDR level (0.05)

We first studied the performance under random signal setting, where the DMPs were randomly distributed. We observed that all the methods controlled the false discovery proportion close to or under the target level (5%) across settings (Fig. 2a). As expected, BH procedure was more conservative than ST procedure when the signal was dense. IHW was generally the most conservative, especially in the dense signal setting. AdaPT was severely conservative in the sparse signal and less informative covariate setting. CAMT was also conservative in that setting, but to a lesser degree. In terms of statistical power, all the covariate-adaptive methods performed better than or similar to the BH/ST procedure when the covariate was informative, indicating their ability to exploit the covariate information (Fig. 2b). CAMT and AdaPT were more powerful than other methods when the signal was dense, or the covariate was highly informative.

However, when the signal was sparse and the covariate was less informative, AdaPT was significantly less powerful than other methods. In contrast, FDRreg and BL performed slightly better than or similar to ST across settings while the performance of IHW varied according to the underlying signal density. IHW was less powerful than FDRreg and BL when the signal was dense, and even slightly less powerful than ST when the covariate was not very informative. As the signal became sparser, IHW was more powerful than FDRreg and BL. We also simulated correlated signals, where the DMPs tended to cluster together. Nevertheless, the pattern remained similar, indicating the robustness of these methods to correlated EWAS signals (Additional file 6: Figure S5). Taken together, all these covariate methods could control the FDR for EWAS data, and their optimal power depended on the specific signal structure as well as the informativeness of the covariate. The favorable performance under the sparse signal makes CAMT and IHW promising candidates for EWAS data since the sparse signal scenarios are statistically more challenging.

### Improved detection power of covariate-adaptive FDR control methods on real EWAS datasets

**Fig. 3:**
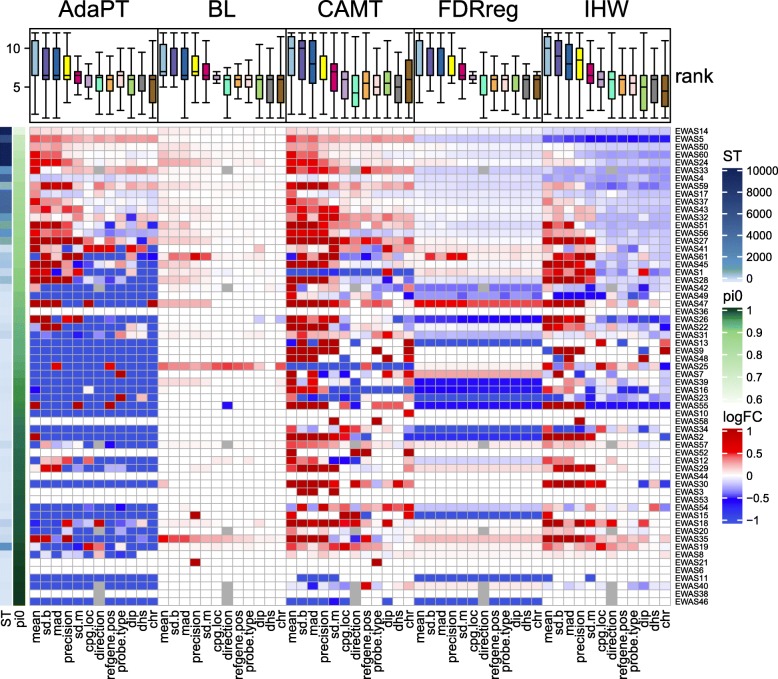
Performance comparison of covariate-adaptive FDR control methods on real EWAS data. Comparison of the detection power across datasets, covariates, and methods (computed as log2 fold change (logFC) to the ST method, all numbers were added a pseudo-count of 1 to avoid 0s). The two left sidebars indicate the number of DMPs detected by ST with a target FDR level 5% and the estimated signal density (pi0), respectively. Rows are ordered by the signal density and columns are ordered by the method and covariate. Top panel shows the rank distribution of each covariate across datasets. The covariates are ranked based on the number of detected DMPs on each dataset. The mean value is used if ranks are the same, and a higher rank indicates higher detection power

We next applied IHW, CAMT, AdaPT, FDRreg, and BL to the 61 real EWAS datasets with the 14 aforementioned covariates in addition to the BH and ST procedure. We first compared the run time of these covariate-adaptive methods with different covariates. IHW was almost an order of magnitude faster than the next fastest method (BL) while FDRreg and AdaPT were computationally the most intensive (Additional file 6: Figure S6). The run time of CAMT and AdaPT was sensitive to the number of categories for categorical covariates (e.g., “dhs” and “chr”) while other methods were less so. In terms of detection power, the ST procedure dominated the BH procedure and never detected less DMPs (Additional file 6: Figure S7). We thus used the ST procedure as the baseline and computed the log fold change in the number of detected DMPs between a method-covariate combination and ST. Figure 3 gave an overview of the detection power for all method-covariate combinations across all datasets, ordered by the estimated signal density. Clearly, none of the methods dominated ST and power loss could occur for all methods when the covariate was uninformative, or the signal density was very low. When the signal density was high (Fig. 3, top), AdaPT and CAMT could significantly improve the detection power with informative covariates and lost little power for less informative ones. BL was slightly more powerful than ST, while FDRreg was in the opposite direction. IHW, on the other hand, was significantly less powerful than ST for most covariates. However, when the signal became less dense (Fig. 3, bottom), CAMT and IHW were overall more powerful than other methods when appropriate covariates were used. In contrast, AdaPT suffered a significant power loss on a number of datasets, regardless of the covariates used. Power loss was also observed for FDRreg; however, to a lesser degree than AdaPT. Interestingly, BL was relatively robust and did not lose power in these situations, but the power improvement was also not extensive (Additional file 6: Figure S8A). Overall, the results on the real data agreed well with those from simulations.

We next ranked the detection power of the covariates on each dataset for each method separately (1—the worst, 12—the best). Among statistical covariates, “mean,” “sd.b,” “mad,” and “precision” outperformed others (Fig. 3, top panel), consistent with the omnibus test results (Fig. 1b). The covariate “mean” was the most informative and ranked top for all methods. Even in datasets such as EWAS33, where there was no obvious correlation between the *p* value and covariates measuring the variance (“sd.m”, “sd.b”, “mad”), a stronger correlation was observed for the covariate “mean” (Additional file 6: Figure S9). Although biological/technical covariates’ performance was not as universal, they could improve detection power for specific datasets. For example, “cpg.loc” was highly informative for EWAS19 and could improve the detection power for all methods. Therefore, selecting the most relevant covariate(s) is important to achieve optimal performance.

In terms of the detection power with the best covariate, CAMT, followed by IHW, had apparent advantages over other methods (Additional file 6: Figure S8B, S10) with little power loss, indicating its potential to improve the detection power for underpowered studies. Interestingly, the omnibus test *p* values of the covariates (Fig. 1b) coincided well with the improvement in detection power across methods (Additional file 6: Figure S11), suggesting that the omnibus test could be potentially used to select the most informative covariate before applying those covariate-adaptive methods. Additional file 6: Figure S10 presented the heat map based on the most significant covariate. Besides using the most informative covariate, combining all informative covariates is also possible for methods that accommodate multiple covariates such as CAMT. We tried both strategies using CAMT. We could see that both approaches achieved quite robust results (Additional file 6: Figure S12), the power improvement (top panel) was similar to or slightly better than the best covariate “mean”.

### Further validation of the detected DMPs by covariate-adaptive FDR methods

The covariate-adaptive FDR methods improved the detection power over traditional methods using informative covariates, and their results covered the majority of the BH detected DMPs (Additional file 3: Table S3). In this section, we further demonstrated the credibility of the improved detection power from different perspectives using several example datasets. We will focus on CAMT and IHW since they have the overall best performance in the simulation studies.

### Down-sampling analysis

**Fig. 4:**
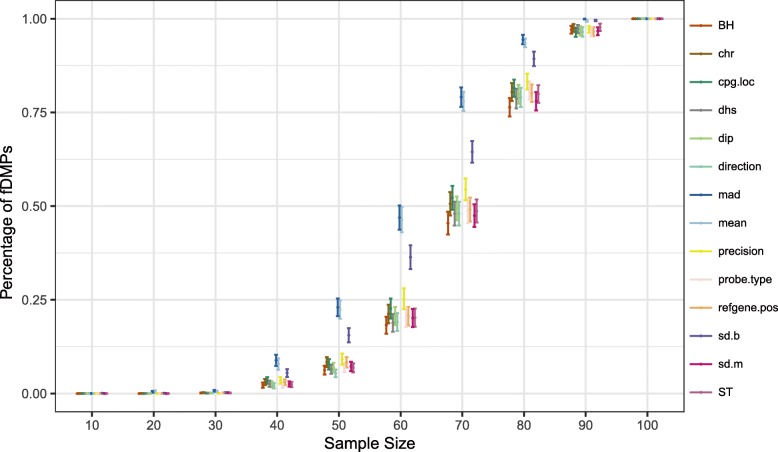
Covariate-adaptive FDR control methods increase the detection power based on the down-sampling analysis. The results are based on the CAMT method. *Y*-axis shows the percentage of fDMPs (DMPs using the *full* dataset) recovered at sample size 10, 20, 30, 40, 50, 60, 70, 80, 90, and 100 for each group. Error bar indicates the standard error

The objective of the down-sampling analysis is to see whether the covariate-adaptive methods have improved power to detect the DMPs based on the full dataset (denoted as “fDMPs”) at a smaller sample size. To achieve this end, we down-sampled a large dataset EWAS51 (*n* = 216, human fetal alcohol spectrum disorder) [52] and performed CAMT with different covariates on the down-sampled datasets. We first defined a list of “gold standard” fDMPs by applying Bonferroni correction (*α* = 0.05) to the association *p* values based on the full dataset. We then compared the percentage of these fDMPs detected by CAMT with different covariates to BH/ST at different sample sizes (Fig. 4). None of the procedures could detect any fDMPs when the sample size was too small (*n* < 30). Significant differences in detection power were observed starting from *n* = 40, and peaked at *n* = 70. As the sample size increased continuously, the power differences became smaller and reached almost 100% for all methods at *n* = 100. Therefore, covariate-adaptive FDR methods could be particularly helpful for a moderately powered study (i.e., power neither too low nor sufficiently high). Notably, covariates “mean,” “mad,” and “sd.b” outperformed other covariates for this dataset. Similar results can be found for IHW, where we observed a similar trend (Additional file 6: Figure S13).

### Detection of age-associated DMPs

**Fig. 5:**
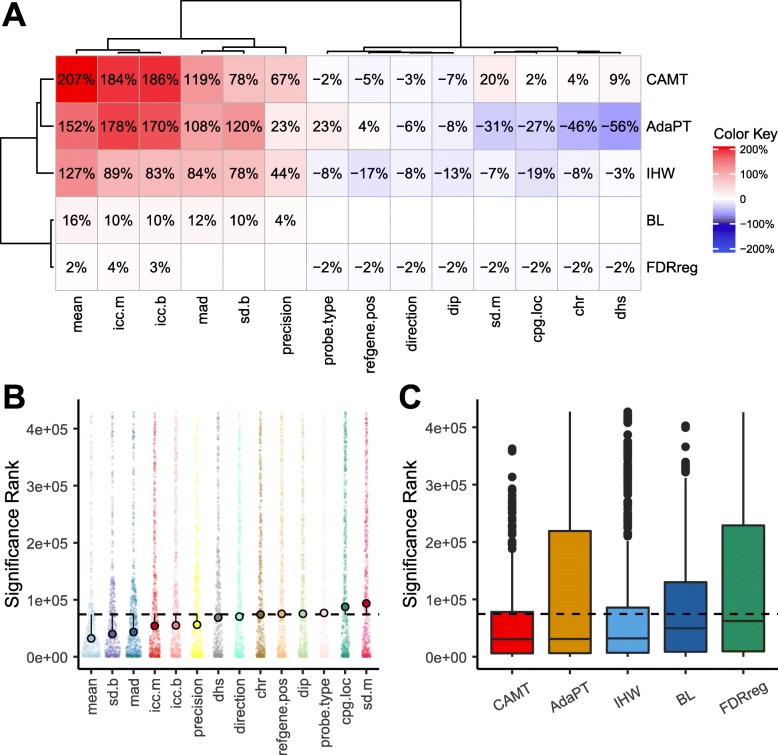
Covariate-adaptive FDR control methods increase the power to detect age-associated DMPs. **a** Percent changes in detected DMPs from the ST method for different method-covariate combinations. Changes with more than 1% are displayed in corresponding cells. **b** Distribution of the significance rank of the gold standard age-associated DMPs (aDMPs) for different covariates using the IHW method. The circle represents the median rank for the specific covariate. A lower rank indicates a more significant result. The dashed line indicates the median rank for the ST method. **c** Distribution of significance rank of the aDMPs for different methods using the methylation “mean” as the covariate

To see whether the covariate-adaptive FDR methods have a better power to detect age-associated DMPs (denoted as “aDMPs”), we defined a list of “gold standard” aDMPs (*n* = 583) derived from two independent studies based on purified blood cell types as described in [46]. We then performed a detailed analysis based on EWAS45, an age EWAS using the peripheral whole blood [25]. For this dataset, all the methods detected more DMPs than ST using informative covariates (Fig. 5). FDR adjustment by the covariate “mean” led to the highest detection power followed by these ICC and variance-based covariates. CAMT, AdaPT, and IHW were overall more powerful than FDRreg and BL with the same informative covariate. We next focused on the list of gold standard aDMPs and compared the distribution of their significance rank among all probes for different method-covariate combinations. We would expect a much lower rank if a procedure was efficient in detecting aDMPs. Based on IHW, a much lower rank for aDMPs was observed using covariates “mean,” “sd.b,” and “mad,” which are the covariates that also yielded high detection power (Fig. 5a, b). A similar pattern was observed for other methods (Additional file 6: Figure S14). Notably, the covariate “mean” achieved the best rank for all the methods. We thus further compared the rank distribution of aDMPs for the five FDR-adaptive methods using the covariate “mean” (Fig. 5c). IHW, AdaPT, and CAMT achieved a significantly lower median rank than other methods, indicating their improved ability to enrich aDMPs with an informative covariate. We repeated the same analysis on another age EWAS dataset (EWAS27) [53] and arrived a similar conclusion (Additional file 6: Figure S15). Taken together, adaptive FDR control with informative covariates not only increased detection power but also improved the power to retrieve biologically relevant DMPs.

### Detection of smoking-associated DMPs

Smoking has been tightly related to DNA methylation change [54–56]. Here we re-analyzed the dataset from an EWAS of smoking [57] (EWAS20) using IHW and CAMT. The majority of the 30 smoking-associated DMPs (denoted as “sDMPs”) reported by [57] were reproduced in our results (Additional file 4: Table S4A). Compared with BH/ST, which produced the same results on this dataset, IHW detected more sDMPs with covariates “sd.b”, “mean,” “mad,” “precision,” and “refgene.pos” (Table 3). Some of these new findings were reported in both European and Chinese populations, including cg19827923, cg19254163, cg07986378, cg15159987, and cg22132788 [58]. Two of the sDMPs (cg04885881 and cg22132788) were also among the list of smoking-related CpGs reported at least three times in a systematic review [59].

**Table 3:** Additional smoking-associated DMPs detected by IHW

**mean**	**mad**	**sd.b**	**precision**	**refgene.pos**
cg04885881^#^	cg04885881^#^	cg04885881^#^	cg19827923*	cg06972908
cg12856965	cg14179389	cg22740783	cg19719391	
cg26337070	cg22740783	cg19827923*	cg07986378*	
cg03147185	cg26337070	cg19719391	cg14712058	
cg13185177	cg19827923*	cg17924476	cg15159987*	
cg19719391	cg19719391	cg08149865		
cg00300637	cg17924476	cg17476951		
cg17924476	cg22132788*^#^	cg19254163*		
cg24688690	cg08149865	cg07986378*		
cg03774957	cg02526790	cg18092474		
cg08149865	cg17476951	cg06972908		
cg17476951	cg13151811	cg08149682		
cg19254163*	cg07986378*	cg14712058		
cg07986378*	cg08730245	cg15159987*		
cg08730245	cg18092474			
cg10322443	cg01207684			
cg18092474	cg06972908			
cg01207684	cg25683268			
cg16519923	cg08149682			
cg06972908	cg14712058			
cg25683268	cg15159987*			
cg15159987*				

* reported in both European and Chinese population

# reported at least three times

In addition, cg00300637, cg17924476, and cg24688690 recovered by the covariate “mean” were all located in the body of AHRR, a gene which encodes aryl-hydrocarbon receptor repressor and is known to be associated with smoking [60, 61]. Moreover, we noted that cg18092474, which was discovered by covariates “sd.b,” “mean,” and “mad” at the same time, was located 1500 bp upstream of the transcription start site (TSS) of the CYP1A1 gene, which encodes a member of the cytochrome P450 superfamily of enzymes and was reported to be associated with maternal smoking in newborns [62–64]. Here we show that smoking is also associated with CYP1A1 methylation in adults’ peripheral blood. The mean methylation level at cg18092474 for both former and current smokers was significantly lower than never smokers (Additional file 6: Figure S16). Polycyclic aromatic hydrocarbons (PAHs), products of cigarette smoking, were shown to induce the expression of CYP1A1 [65]. Therefore, we speculate that PAHs may upregulate the expression of CYP1A1 by hypomethylation at the cg18092474 position. These results were also supported by CAMT (Additional file 4: Table S4B), which had substantial overlap with the IHW results (Additional file 6: FigureS17).

### Potential of using EWAS *p* value from a related disease/phenotype as a covariate

**Fig. 6:**
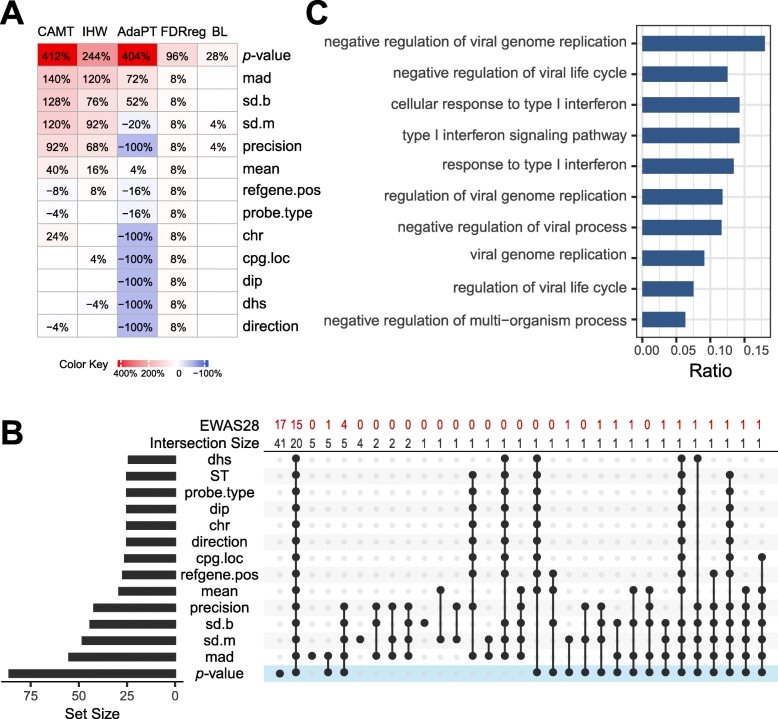
The use of the association *p* value from a related study as a covariate. The *p* values from an EWAS of systemic lupus erythematosus (SLE) using CD19+ B cells (EWAS28) were used as the covariate to adjust the *p* values for another EWAS of SLE using CD4+ T cells (EWAS29). **a** Percent changes in detected DMPs from the ST method for different method and covariate combinations. Changes with more than 1% are displayed in corresponding cells. **b** Intersections in detected DMPs for the IHW method using different covariates. The connected dots indicate the intersected covariates. Left sidebar indicates the number of DMPs detected by the IHW method using different covariates. As a comparison, the ST method is also included. The intersection size as well as the number of DMPs detected on EWAS28 is indicated on the top. **c** Top 10 GO terms from enrichment analysis based on the IHW method using the *p* value from a related study as the covariate. The *x*-axis indicates the percentage of DMPs in the enriched biological process (out of the total DMPs). And GO terms from top to bottom are arranged in descending order (top 1 to top 10)

When there are published EWAS data from a similar disease or the same disease but from a different tissue or cell type, we may want to use the published data to improve the detection power of a new EWAS since we expect signal sharing across datasets. Covariate-adaptive FDR methods provide a convenient way to exploit previous data by using the association *p* value as a covariate. To investigate such possibility, we performed analysis based on EWAS28 and EWAS29, two datasets from EWAS of systemic lupus erythematosus (SLE) using CD19+ B cells and CD4+ T cells, respectively [66]. The signal in EWAS28 (539 DMPs) was much stronger than that of EWAS29 (26 DMPs) according to results from ST. Thus, we used the *z*-score transformed *p* value from EWAS28 as the covariate to adjust the *p* value of EWAS29 to see whether we can improve the detection power of a less-powered study. We first verified the assumption of independence under the null using stratified histograms (Additional file 6: Figure S18). Next we applied the omnibus test for assessing the informativeness. The omnibus test showed the new covariate “*p* value” was highly informative (*p* = 0.002), and it appeared to be more informative than the previously studied covariates for all the covariate-adaptive methods (Fig. 6a). Application of IHW with the new “*p* value” covariate detected far more DMPs than other covariates (Fig. 6b). Remarkably, it recovered many DMPs detected by EWAS28 but otherwise missed by EWAS29 alone (Fig. 6b). According to Gene Ontology (GO) enrichment analysis based on the genes detected using the new covariate, three out of the top five GO enrichment terms belonged to type I interferon (INF I)-related biological processes (Fig. 6c, all *p* value < 1E−20), which plays an important role in SLE and serves as a therapeutic target [67]. Although these terms were also enriched in the results of the ST method, they were not among the top 10 terms (Additional file 5: Table S5). Similar results can be found by applying CAMT (Additional file 6: Figure S19A&B). Collectively, the use of *p* value from a related EWAS as the covariate in FDR control serves as a convenient way to integrate previous knowledge to enrich signal and is a promising approach to an underpowered study.

## Discussion

In this study, we explored the use of start-of-the-art covariate-adaptive FDR control methods in multiple testing correction for epigenome-wide association testing. We also studied the performance of various EWAS-related covariates based on a large collection of EWAS data. We show that all these covariate-adaptive methods could control the false discovery rate at the target level and were substantially more powerful than traditional FDR control methods such as Benjamini-Hochberg’s step-up procedure [28] and Storey’s *q* value procedure [29], once informative covariates were used. Compared to the benchmarking simulation study in [44], which simulated the *p* values directly, we simulated original methylation data, upon which *p* values were calculated. In this way, we preserved the typical correlation structure among *p* values observed in real EWAS data, and thus the evaluation of false discovery rate control was more rigorous. In addition, we propose an omnibus test for assessing the informativeness of the covariates, investigate the performance of various EWAS-relevant covariates, and benchmark the methods on a large number of real EWAS datasets.

For the 61 real EWAS datasets, the most informative covariate could improve the detection power by a median of 68% and 25% for CAMT and IHW, the two best performing methods, compared to the ST procedure. Remarkably, for 9 EWAS datasets, where BH/ST procedure did not make any discovery, CAMT and IHW were able to make positive findings in four and one of the datasets using informative covariates, respectively. We further demonstrated with concrete examples that the additional DMPs recovered were biologically meaningful and could help reveal more biological insights. Given the enormous cost associated with epigenome-wide profiling, using covariate-adaptive multiple testing could potentially rescue an underpowered study, reduce the discovery cost, and ultimately shorten the bench to bedside cycle time.

Although the investigated covariate-adaptive FDR control methods all maintained the target FDR level, their power varied tremendously depending on the covariate informativeness and signal density. CAMT and AdaPT were most powerful when the signal density was high or the covariate was strongly informative, suggesting their strong ability to utilizing the underlying information. However, when the signal became sparser and the covariate less informative, AdaPT suffered from a great power loss, which was consistent with the observation in [45]. Although some remedy could be possibly invoked to compensate the power loss as suggested in [45], no implementation has been available in the AdaPT package as of January 2020. On the other hand, CAMT was less susceptible to such power degradation, although some power loss was still observed for less informative covariates in real data. On the contrary, IHW had the best performance when the signal was sparse, but the performance deteriorated with increasing signal density. In circumstances where the signal is dense, and the covariate is not very informative, IHW could be less-powerful than ST. The power of FDRreg and BL, on the other hand, were overall more similar to ST. Significant power gain was only observed when the signal was relatively dense, and the covariate was very informative. In the real data, BL was more robust than FDRreg. Although there was no uniformly most powerful procedure, CAMT and IHW stood out due to their excellent performance under the sparse signal setting, where power improvement is most needed. Also, IHW was an order-of-magnitude faster than other methods, making it particularly appealing for large-scale analyses.

All the covariate-adaptive FDR control methods could lose power if the covariate was not informative. Thus, assessment of the informativeness of the covariate was recommended before applying a covariate-adaptive method. The traditional way of assessment relied on statistical graphics such as scatter plots or stratified *p* value histograms [40]. Such an assessment was not effective when the signal was sparse. We thus developed a powerful omnibus test to test the dependence between the *p* value and the covariate accounting for potential nonlinearity and signal sparsity. We show that the omnibus test *p* value correlated well with the power improvement for various covariate-adaptive methods. However, the optimal omnibus *p* value cutoff to achieve significant power gain differed by method and simulation studies may help determine the best cutoff.

The validity of these covariate-adaptive methods depends on the assumption that the *p* value is independent of the covariate under the null. This assumption is usually assessed empirically by stratified histograms: for large *p* values (e.g., *p* values ≥ 0.5), the distribution should appear uniform across strata. A variant of the omnibus test could be used to formally assess the assumption of independence under the null. Specifically, the omnibus test can be focused on the large *p* values (*p* value > 0.5, considered to be dominantly null *p* values) and tailor the *p* value cutoff points to be more equally spaced (e.g., *p*^(*i*)^ ∈{0.1, 0.2, 0.4, 0.6, 0.8, 0.9}-quantile of the *p* values). We show by additional simulations (Additional file 6: Figure S20, simulation details in the legend) that this test is very powerful and can detect a very moderate deviation from the assumption. Therefore, in practice, we could apply both types of omnibus test simultaneously and select covariates that are significant in the original test (i.e., overall dependency between *p* value and covariate) and not significant in the suggested variant (i.e., the dependency is not due to violation of the independence assumption under the null).

For EWAS data, we investigated 14 potential useful covariates. We found that statistical covariates are more informative than biological/technical covariates, as the former are more directly related to statistical power. In addition, statistical covariates could also be informative of the prior null probability. For example, the variance of the feature is considered to be a good indicator of the null probability [40] since an invariant feature is less likely to be differential. Variance-based filters have been frequently used to exclude invariant genomic features to reduce multiple testing burden [68] and increase statistical power. One advantage of using covariate-adaptive methods is that filtering is no longer necessary, and the method could automatically down-weight these less likely features according to their variances. Interestingly, “sd.b” performed better than “sd.m” on the real data even though the association tests were performed on the M-values. This could be explained by the fact that “sd.b” as the standard deviation on the Beta-value might be a more biologically relevant measurement of methylation variability and is more informative of the null probability. The strong performance of the covariate “mean” is somewhat unexpected and may be explained by two reasons, which are related to the prior null probability and statistical power, respectively. First, DMPs are enriched in certain regions such as the promoters [30, 31], where the methylation levels are similar—either low or high (Additional file 6: Figure S3A). Second, the statistical power to detect DMPs in the middle of the methylation spectrum is higher than those in the two ends, where the methylation changes are constrained (Additional file 6: Figure S3B). The covariate “dip” was the least informative among all statistical covariates since the majority of CpGs, including those DMPs, have unimodal distributions and the dip statistic is not very informative. Compared to statistical covariates, external covariates were less universal than statistical covariates, and they were informative only for specific datasets. For instance, the covariate “cpg.loc” was very powerful for EWAS19 since DMPs in pancreatic ductal adenocarcinoma were enriched in CpG islands [20].

Besides the traditional covariates, we also explored the use of *p* value from the same or similar disease/phenotype as a covariate, and we demonstrated a substantial power gain. Using *p* value as a covariate provides a convenient way to conduct integrative analysis without the need for sophisticated statistical modeling or access to the raw data. Other possible covariates, which are not explored here, include the detection *p* value, existence of SNP in the probe, possible cross-hybridization [69] and probe-level reproducibility [70] from an independent study. With large-scale genome annotation endeavors such as the ENCODE project [71], an increasing number of genomic annotations are now available and they can all be used as potential covariates. The proposed framework can be used to evaluate their relevance for EWAS.

Finally, as several covariates may be informative for a particular dataset, finding the optimal combination of them will be an interesting topic. Except for IHW, all the covariate-adaptive methods can accommodate multiple covariates. Coupling the omnibus test and these methods may be a feasible solution.

## Conclusions

Covariate-adaptive FDR control methods can significantly increase the detection power for EWAS using informative covariates. The choice of the optimal method depends on the underlying signal density and the informativeness of the covariate. The informativeness of the covariate can be assessed based on the proposed omnibus test. For a dense signal, CAMT and AdaPT are the most powerful; for a sparse signal, CAMT and IHW are most promising.

## Methods

### Data

We collected 58 methylation datasets from GEO and split them into 61 EWAS datasets according to the phenotype, tissue source, and cell type under the following criteria: (1) the platform is Infinium Human Methylation 450K BeadChip; (2) sample size is no less than 100. Note that the majority of the 61 EWAS datasets are for different outcomes with some for the same outcome such as smoking. For datasets with raw IDAT files (the raw intensity data from the beadchip) available, R function *minfi::read.metharray.exp* (version 1.26.2) [72] was used to extract Beta-values and detection *p* values. Otherwise, we obtained the data from the soft file on GEO via *GEOquery::getGEO* (version 2.48.0) [73]. The annotation file of Infinium Human Methylation 450K BeadChip was obtained from R package *IlluminaHumanMethylation450kanno.ilmn12.hg19* (version 0.6.0) [74].

### Quality control

The normalization of raw data was carried out by *preprocessQuantile* [75] function from *minfi* package. Samples with mean detection *p* value greater than or equal to 0.01 were removed, and probes with median detection *p* value across all samples less than or equal to 0.01 were filtered. Probes designed for sex chromosomes or autosomal probes co-hybridizing to sex chromosomes (cross-reactive) [69] were also filtered. Besides, probes with common SNPs either at the CpG interrogation site or at the single nucleotide extension site were excluded, regardless of minor allele frequency.

### Differential methylation analysis and multiple testing correction

Before performing multiple testing correction, surrogate variable analysis was conducted using R package *isva* (version 1.9) [76] and *SmartSVA* (version 0.1.3) [46] to capture significant sources of methylation variability such as cellular heterogeneity, age, and other unknown batch effects. The resulting surrogate variables were treated as covariates in the linear regression model to address potential confounding effects of cellular heterogeneity and batch effects. Differential methylation analysis was performed using the function *cpg.assoc* from R package *CpGassoc* (version 2.60) [77] and conducted on M-values [78] (logit.transform = TRUE). The association *p* values were then corrected for multiple testing by various FDR control methods. Traditional FDR control methods Benjamini-Hochberg’s step-up procedure (BH) and Storey’s *q* value procedure (ST) were performed using the R package *stats* (version 3.5.3) [79] and *qvalue* (version 2.12.0) [80], respectively. Covariate-adaptive FDR control methods IHW, CAMT, BL, AdaPT, and FDRreg were performed using R package *IHW* (version 1.8.0) [40], *CAMT* (version 1.0) [44], *swfdr* (version 1.6.0) [42], *adaptMT* (version 1.0.0) [43], and *FDRreg* (version 0.1) [39] with default settings (except *FDRreg*, theoretical null type was used to achieve more robust performance). See details in Table 2. A target FDR level of 5% was used throughout the study.

### An omnibus test for assessing the informativeness of the covariates

The informativeness of the covariate is assessed by testing the association between the *p* value from differential methylation analysis and the covariate. Due to potential sparsity and nonlinearity of the association signal, rank-based tests (e.g., Spearman’s rank test and Kruskal-Wallis test) may not be powerful since the vast majority of the *p* values are not expected to be associated with the covariate under the null. We thus develop an omnibus test, which zooms into the low *p* value region and addresses nonlinearity by categorizing the covariate if it is continuous. Denote *p*_1_, *p*_2_, …, *p*_*m*_ the *p* values for *m* CpGs and *x*_1_, *x*_2_, …, *x*_*m*_ the values for covariate *X*. For a given *p* value cutoff *p*^(*i*)^, *i* = 1, …, *I*, we convert the *p* value into a binary variable *P*^(*i*)^ (≤*p*^(*i*)^, > *p*^(*i*)^). We next consider two scenarios:

1. *X* is categorical. To quantify the association between *P*^(*i*)^ and *X*, we use the *χ*^2^ test and denote *q*^(*i*)^ as the −log *p* value from the *χ*^2^ test. We then define the omnibus test statistic for a categorical covariate as
  $${t}^o=\underset{i}{\max }{q}^{(i)},$$
  which pools information across different dichotomizations.
2. *X* is continuous. We further categorize *X* to address potential nonlinear effects. For a given number of categories *n*^(*j*)^, *j* = 1, …, *J*, we convert *X* into a categorical variable *X*^(*j*)^ with equal category size. To quantify the association between *P*^(*i*)^ and *X*^(*j*)^, we use both *χ*^2^ test and Cochran–Armitage test for trend [81] and denote ${q}_1^{\left(i,j\right)}$ and ${q}_2^{\left(i,j\right)}$ as the −log *p* value from the respective tests. The use of Cochran–Armitage test for trend is to compensate potential power loss of *χ*^2^ test when *P*^(*i*)^ and *X*^(*j*)^ have a monotone relationship. We then define the omnibus test statistic for a continuous covariate as
  $${t}^o=\underset{i,j}{\max}\left\{\max \left[{q}_1^{\left(i,j\right)},{q}_2^{\left(i,j\right)}\right]\right\}.$$

We next use permutation to assess the significance. Denote the test statistic under permutation as ${t}_k^p\ \left(k=1,\dots, K\right)$. The omnibus test *p* value is calculated

$$pval=\frac{1+{\sum}_k\mathrm{I}\left(\ {t}_k^p\ge {t}^o\right)}{1+K}.$$

For both simulated and real datasets, we use *p*^(*i*)^ ∈ {0.001, 0.005, 0.01, 0.05, 0.1, 0.2}-quantile of the *p* values ,*n*^(*j*)^ ∈ {2, 4, 8, 16, 32}, *K* = 999. Since nearby CpGs are usually correlated, to satisfy the exchangeability assumption of the permutation test, we ordered CpGs by their genomic position and down-sampled them to achieve near zero auto-correlation (R *stats::acf*) in real data analysis.

### Simulation studies

#### Simulations for comparing covariate-adaptive FDR control methods

To preserve the correlation structure observed in real data, we based our simulation on a real EWAS dataset [50]. We drew random samples from the control group to create two groups of equal sample size (*n*_1_ = *n*_2_ = 80), based on which we added differential signals. For demonstration purpose, we only used CpGs from the chromosome 13 (*m* = 11, 808). To study the impact of signal density, signal strength, and informativeness of the covariate, we simulated 27 (3 × 3 × 3) settings with a varying degree of signal density (sparse, medium, and dense), signal strength (weak, moderate, and strong), and covariate informativeness (none, moderate, and strong). We first simulated “random signals,” where the differential CpGs were randomly distributed along the chromosome. Specifically, we first generated the covariate *x*_*i*_~*N*(0, 1), *i* = 1, …, *m*. Given *x*_*i*_, we generated *π*_0*i*_, the probability of being the null for the *i*th hypothesis, as

$${\pi}_{0i}=\frac{\exp \left({\eta}_0+c{x}_i\right)}{1+\exp \left({\eta}_0+c{x}_i\right)},$$

where we set *η*_0_ ∈ {3.5, 2.5, 1.5}, representing sparse, medium, and dense signals (3%, 8%, and 18% under no covariate effect), and c ∈ {0, 1, 1.5}, representing a non-informative, moderately informative, and strongly informative covariate, respectively. With *π*_0*i*_, we generated the differential status *H*_*i*_~Bernoulli(1 − *π*_0*i*_). If a CpG was differential, i.e., *H*_*i*_ = 1, we added a methylation difference *f*_*i*_ ∈ {0.27, 0.33, 0.45} (on M-value) to samples from one group, representing a weak, moderate, and strong effect. Linear regression was then performed to generate the association *p* values, which were further analyzed by various covariate-adaptive FDR procedures, along with the covariate. We also simulated “correlated signals,” where the differential CpGs were clustered on the chromosome. To achieve this end, we generated *x*_*i*_ from a autoregressive model (R *stats::arima.sim*, ar = 0.75).

#### Simulations for studying the performance of the omnibus test

We simulated *m* = 10,000 hypotheses and investigated both categorical and continuous covariates. For a categorical covariate, we randomly sampled *x*_*i*_ ∈ {1, 2, 3, 4, 5}, *i* = 1, …, *m*.. For a continuous covariate, we generated *x*_*i*_~*N*(0, 1), *i* = 1, …, *m*. Next, we used the same aforementioned simulation strategy on standardized *x*_*i*_ (mean 0, sd 1) to generate *H*_*i*_, where *η*_0_ ∈ {3.5, 2.5, 1.5}, representing sparse, medium, and dense signals, and *c* ∈ {0, 0.25, 0.375}, representing no dependence, weak dependence, and moderate dependence on the covariate, respectively. To simulate a nonlinear dependency for a continuous covariate, we replaced *x*_*i*_ with ${x}_i^2$ in generating *π*_0*i*_. Next, we generated the *z*-score *z*_*i*_~*N*(0, 1), if *H*_*i*_ = 0, and *z*_*i*_~*N*(2.68, 1) if *H*_*i*_ = 1. We finally generated the *p* value based on 1 − Φ(*z*_*i*_), where *Φ* is the c.d.f of the standard normal.

### Covariates

We investigated 14 covariates “mean,” “sd.b,” “sd.m,” “mad,” “dip,” “precision,” “direction,” “icc.b,” “icc.m,” “refgene.pos,” “cpg.loc,” “chr,” “dhs,” and “probe.type.” The definition, calculation, and representation of these covariates could be found in Table 1. If the covariate was categorical, we coded it as a factor in R and transformed into a model matrix if needed. If the covariate was continuous, we applied natural cubic spline transformation to allow a certain degree of nonlinearity. We used Bayesian information criterion (BIC) to determine the optimal degree of freedom on several real datasets, and a degree of freedom of 6 was found to be adequate to achieve satisfactory results. We thus set the degree of freedom to be 6 in the natural spline (*ns*(x, df = 6) in R package *splines*, version 3.5.0) [79]. Besides, the same covariates were used for both pi0 and f1 if needed (CAMT and AdaPT).

### Down-sampling analysis

We used EWAS51 (112 human fetal alcohol spectrum disorder samples with 104 controls) to perform down-sampling analysis. CpGs with Bonferroni-corrected *p* value below 0.05 based on the full sample size were defined as fDMPs. Since EWAS51 was of binary phenotype, we subsampled each phenotype to a sample size 10, 20, 30, 40, 50, 60, 70, 80, 90, and 100. At each sample size, 100 replications were performed.

### GO and KEGG enrichment analysis

GO and KEGG enrichment analysis was done by *gometh* function of R package *missMethyl* (version 1.16.0) [82]. All CpG labels in the array data were used as background.

### Visualization

For data cleaning and transformation, R package *tidyverse* (version 1.2.1) [83] was applied. Figures were generated and arranged by R package *ggplot2* (version 3.1.1) [84], *ggforce* (version 0.3.0) [85], *ComplexHeatmap* (version 2.1.0) [86], *UpSetR* (version 1.4.0) [87], *ggpubr* (version 0.1.7) [88], and *cowplot* (version 1.0.0) [89]. Clustering heatmaps were generated with distance measure “euclidean” and clustering method “ward.D”.

## Supplementary information

Additional file 1: **Table S1.** Description of the Datasets.

Additional file 2: **Table S2.** Informativeness statistics for the 14 covariates based on the omnibus test.

Additional file 3: **Table S3.** Percentage of BH-significant DMPs recovered by IHW with different covariates.

Additional file 4: **Table S4A.** Reproducing the smoking EWAS results on EWAS20. **Table S4B.** Additional smoking-associated DMPs detected by CAMT.

Additional file 5: **Table S5.** Top 20 GO Terms based on ST Results.

Additional file 6: Supplementary figures.

Additional file 7: Review history.

## Data Availability

All the EWAS datasets analyzed during the current study are publicly available on the Gene Expression Omnibus (GEO, http://www.ncbi.nlm.nih.gov/geo) [90] at corresponding GEO accession provided in Additional file 1: Table S1. The data and source code to reproduce all the figures in this study are available on GitHub repository at the site [91] and Zenodo [92].

## Abbreviations

EWAS: Epigenome-wide association studies
FDR: False discovery rate
FWER: Family-wise error rate
BH: Benjamini-Hochberg step-up procedure
ST: Storey’s *q* value procedure
DMPs: Differentially methylated CpG positions
LFDR: The conditional local FDR
FDRreg: FDR regression
IHW: Independent hypothesis weighting
ASH: Adaptive shrinkage
BL: Boca and Leek’s FDR regression
AdaPT: Adaptive *p* value thresholding
CAMT: Covariate Adaptive Multiple Testing
GEO: Gene Expression Omnibus
ICC: Intraclass correlation coefficient
fDMPs: Differentially methylated CpG positions based on the *full* dataset
aDMPs: Age-associated differentially methylated CpG positions
sDMPs: Smoking-associated differentially methylated CpG positions
TSS: Transcription start site
PAHs: Polycyclic aromatic hydrocarbons
SLE: Systemic lupus erythematosus
INF I: Type I interferon
GO: Gene Ontology
ENCODE: The Encyclopedia of DNA Elements
