# Supplementary material for: Leveraging biological and statistical covariates improves the detection power in epigenome-wide association testing
Source: Genome Biol. 2020 Apr 6;21:88. doi: 10.1186/s13059-020-02001-7 (PMC7132874; doi:10.1186/s13059-020-02001-7)
Supplement: Supplementary file 6 — Supplementary figures. [file 13059_2020_2001_MOESM6_ESM.docx]

**Supplementary Figures**

**
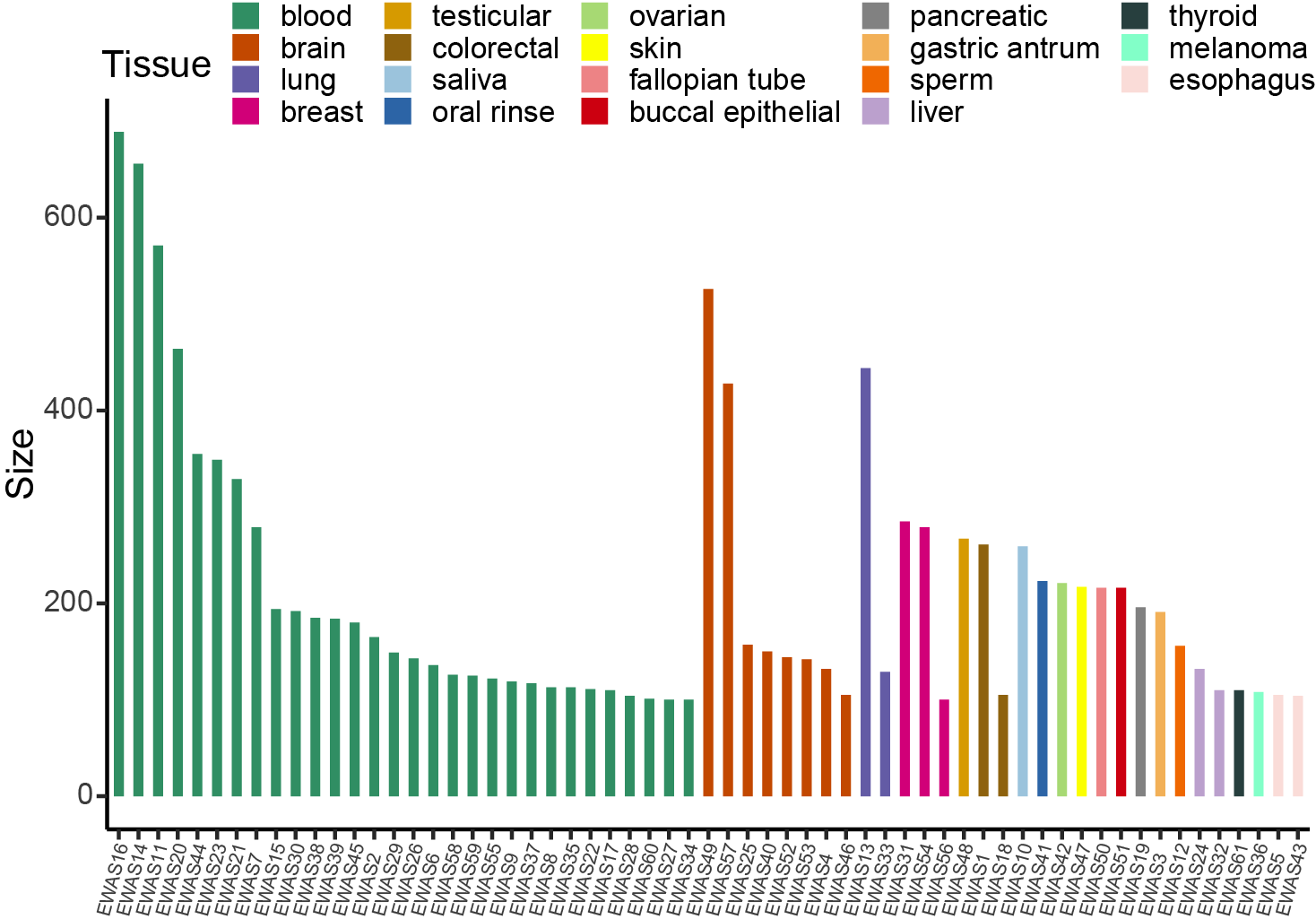
**

**Figure S1 Tissue distribution of the EWAS datasets.** The y-axis displays the sample size for each dataset, and datasets on the x-axis are arranged in descending order by sample size within each tissue source indicated by different colors.

**
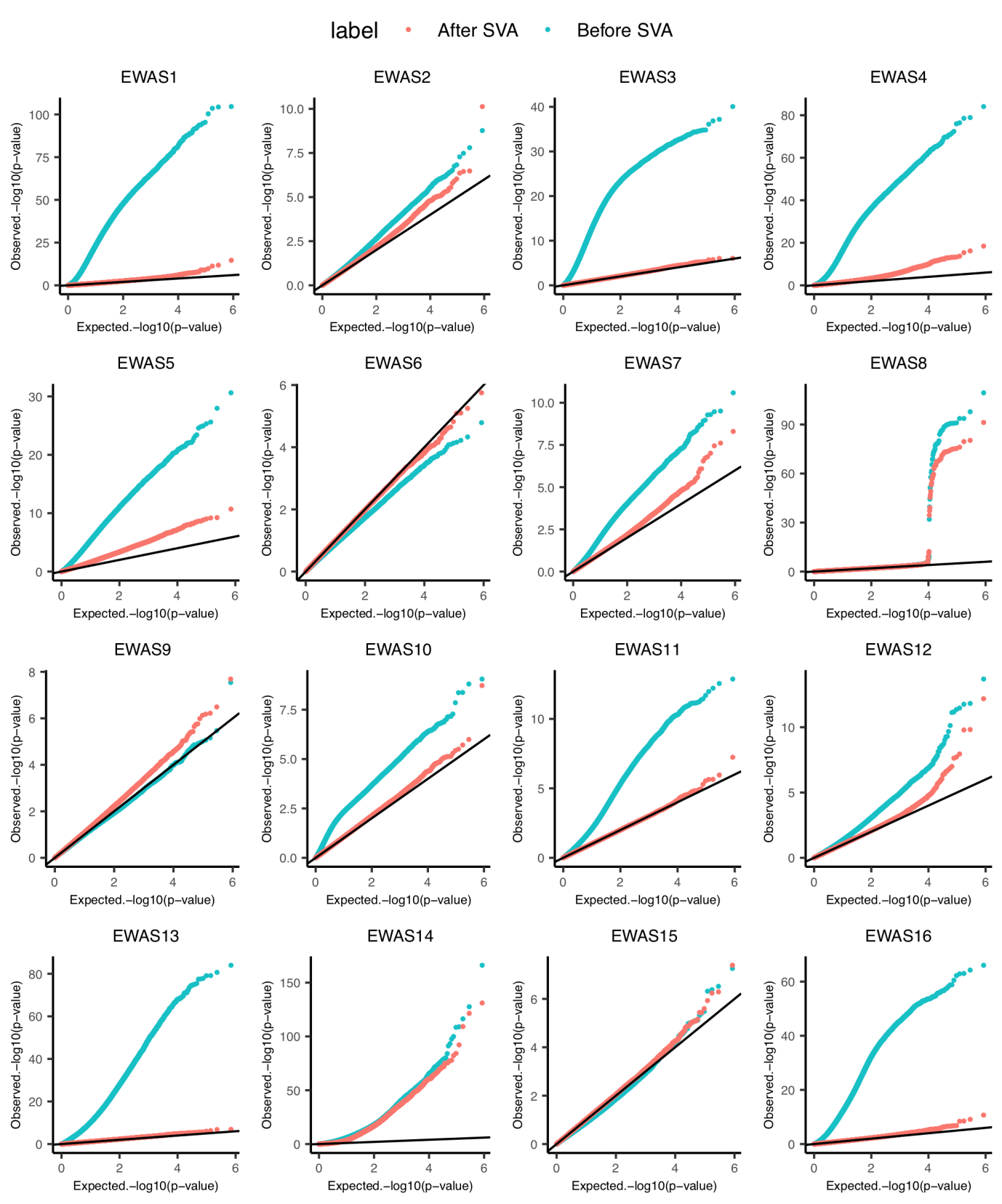
**

**
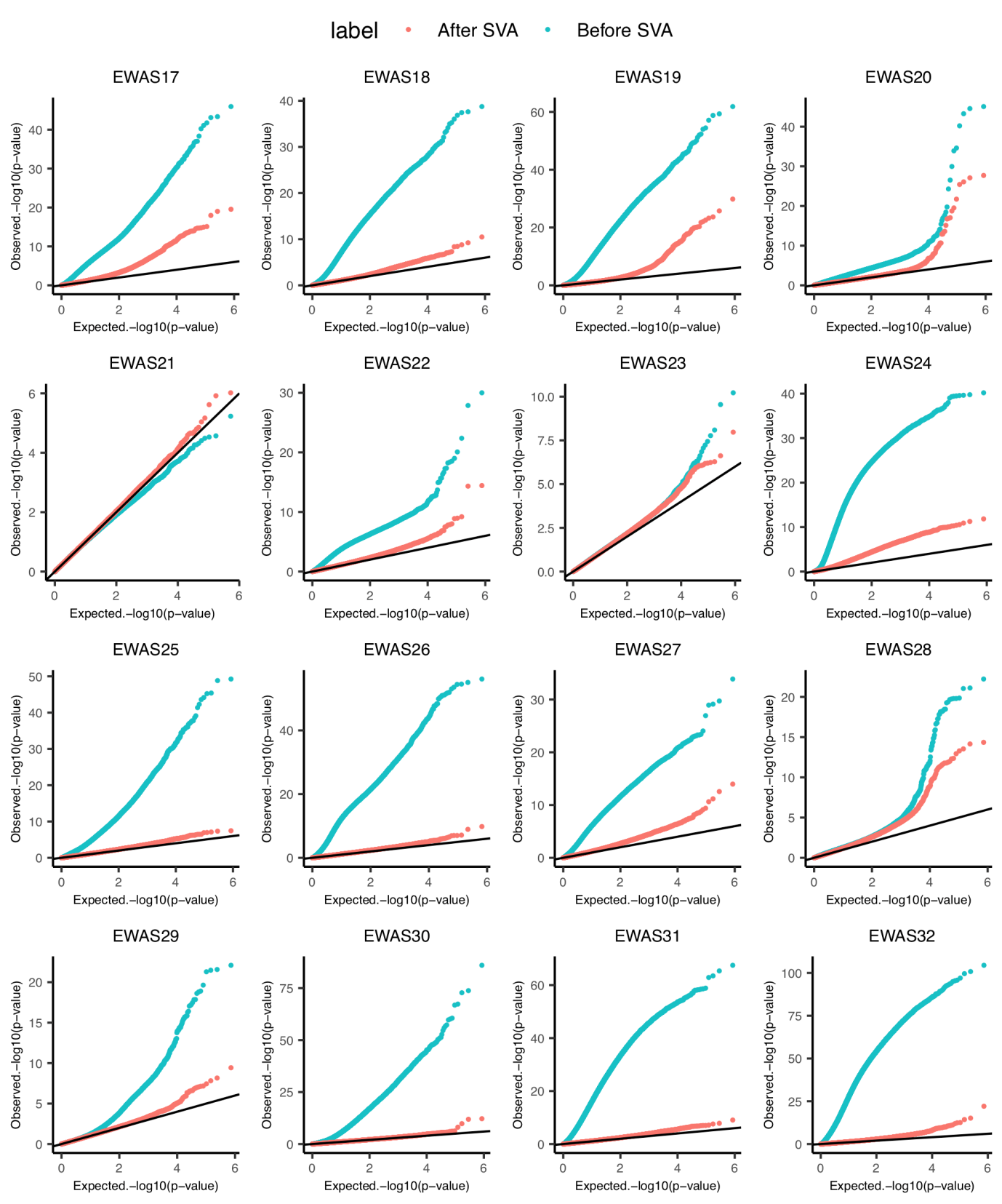
**

**
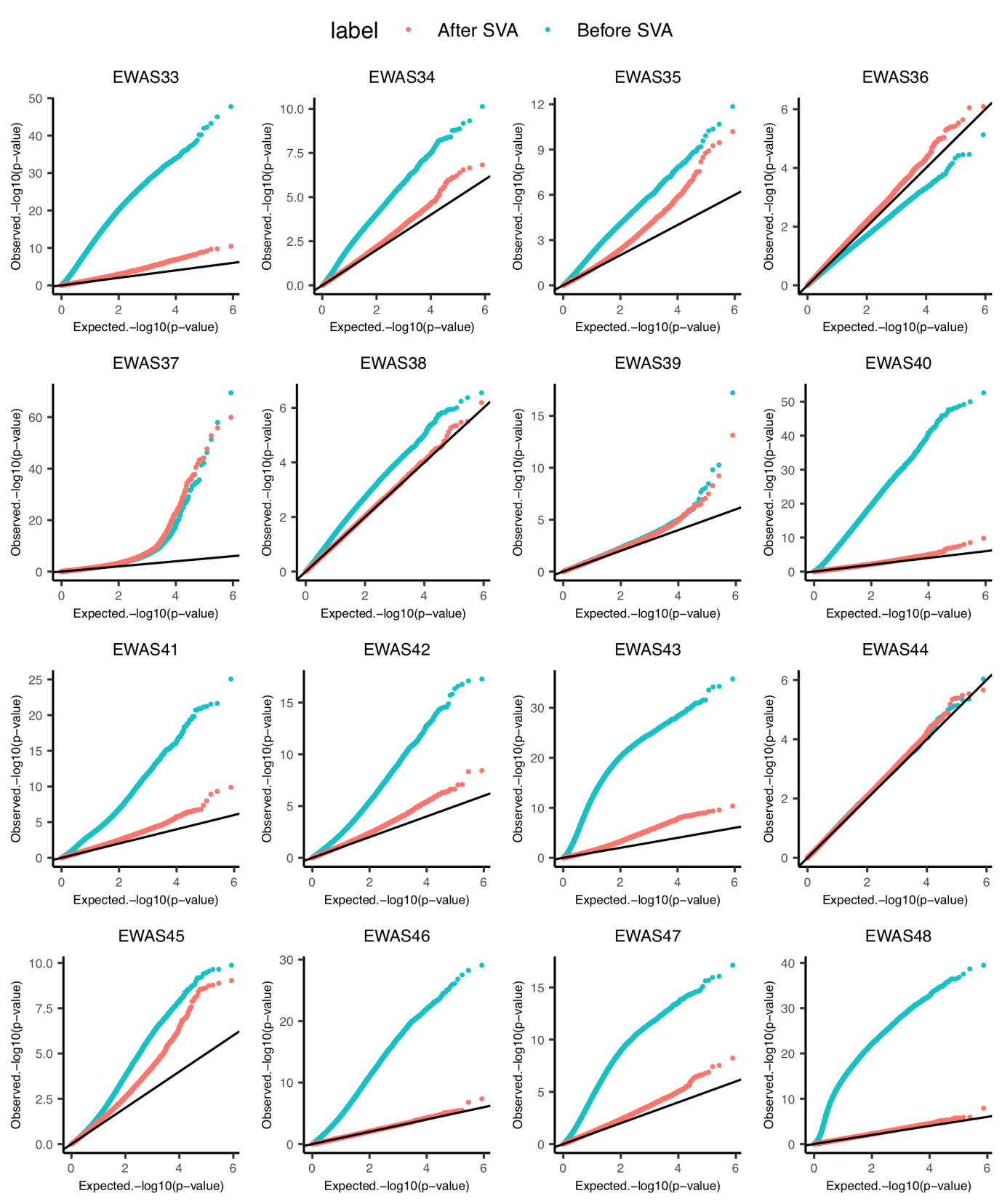
**

**
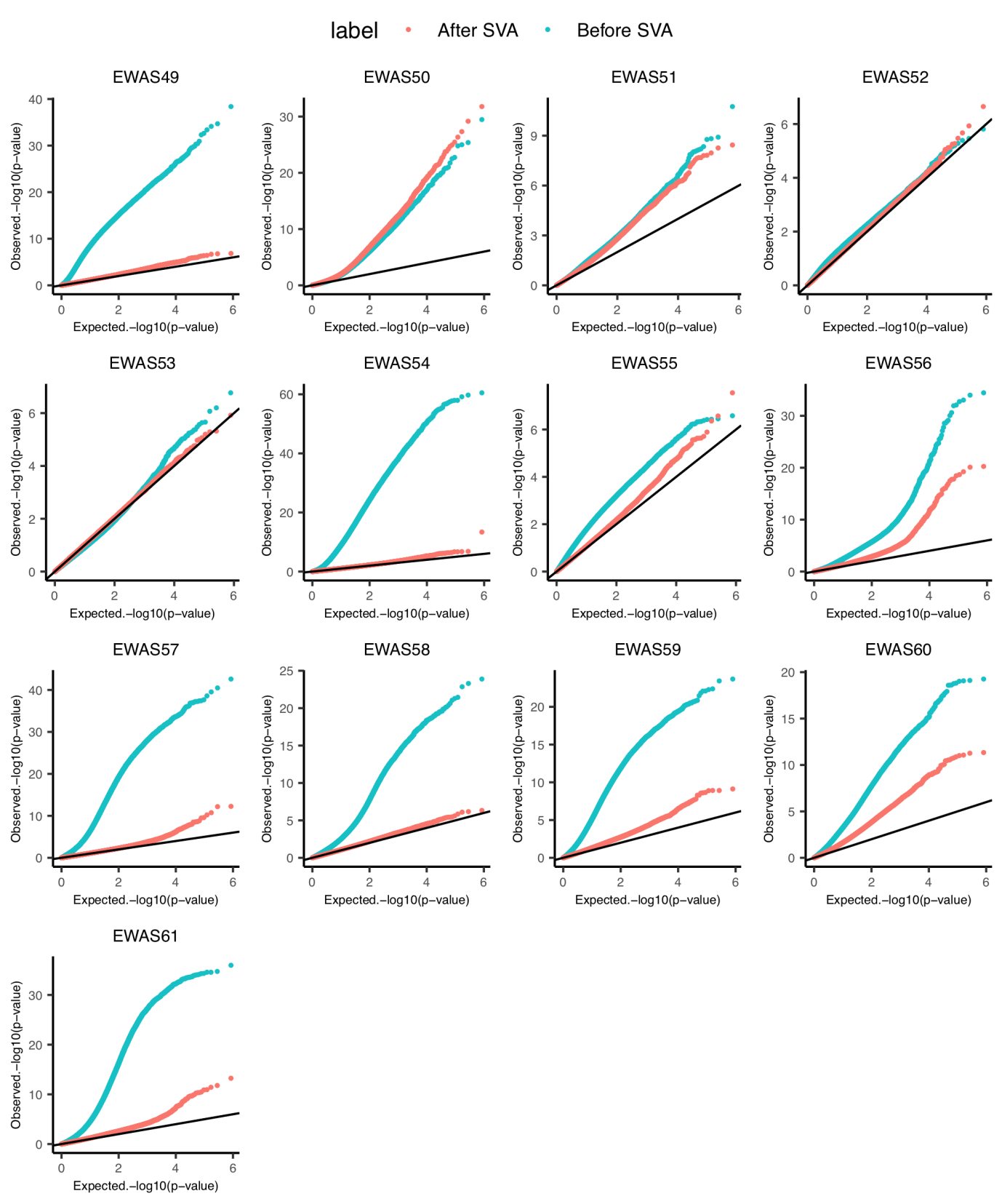
**

**Figure S2** **QQ-plots of expected *p*-values versus observed *p*-values before and after adjustment of surrogate variables for all EWAS datasets**


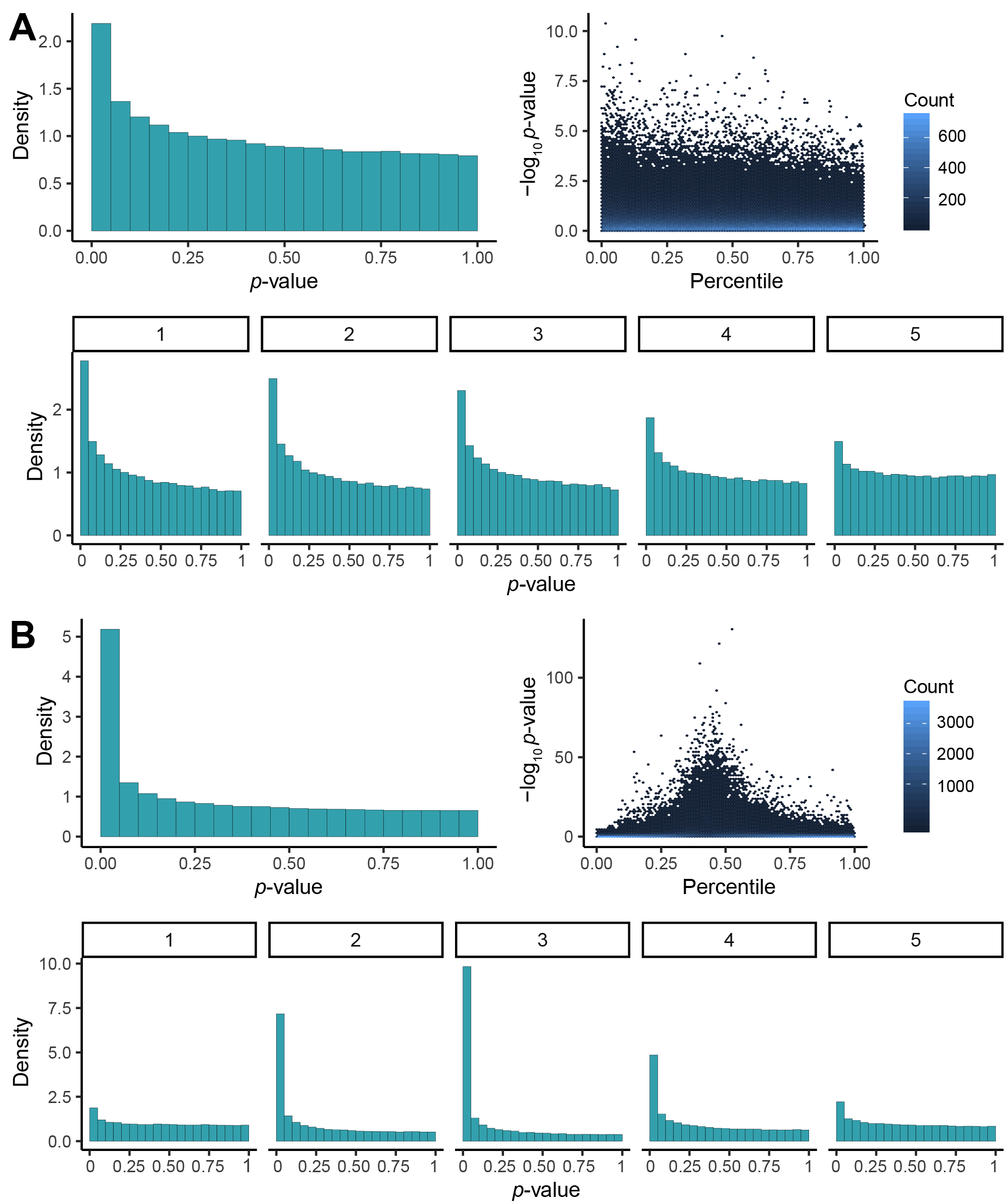


**Figure S3** **Dependency between the *p*-value and the mean methylation ("mean")**. Two examples are shown: (A) low *p*-values are enriched in low methylation region and (B) low *p*-values are enriched in mid methy­­lation region. For each example, the top left panel shows the histogram of all raw *p*-values. The top right panel shows the scatter plot of the -log(*p*-value) against the percentile of the covariate "mean". Bottom shows the histogram stratified by the quintiles of the covariate "mean".


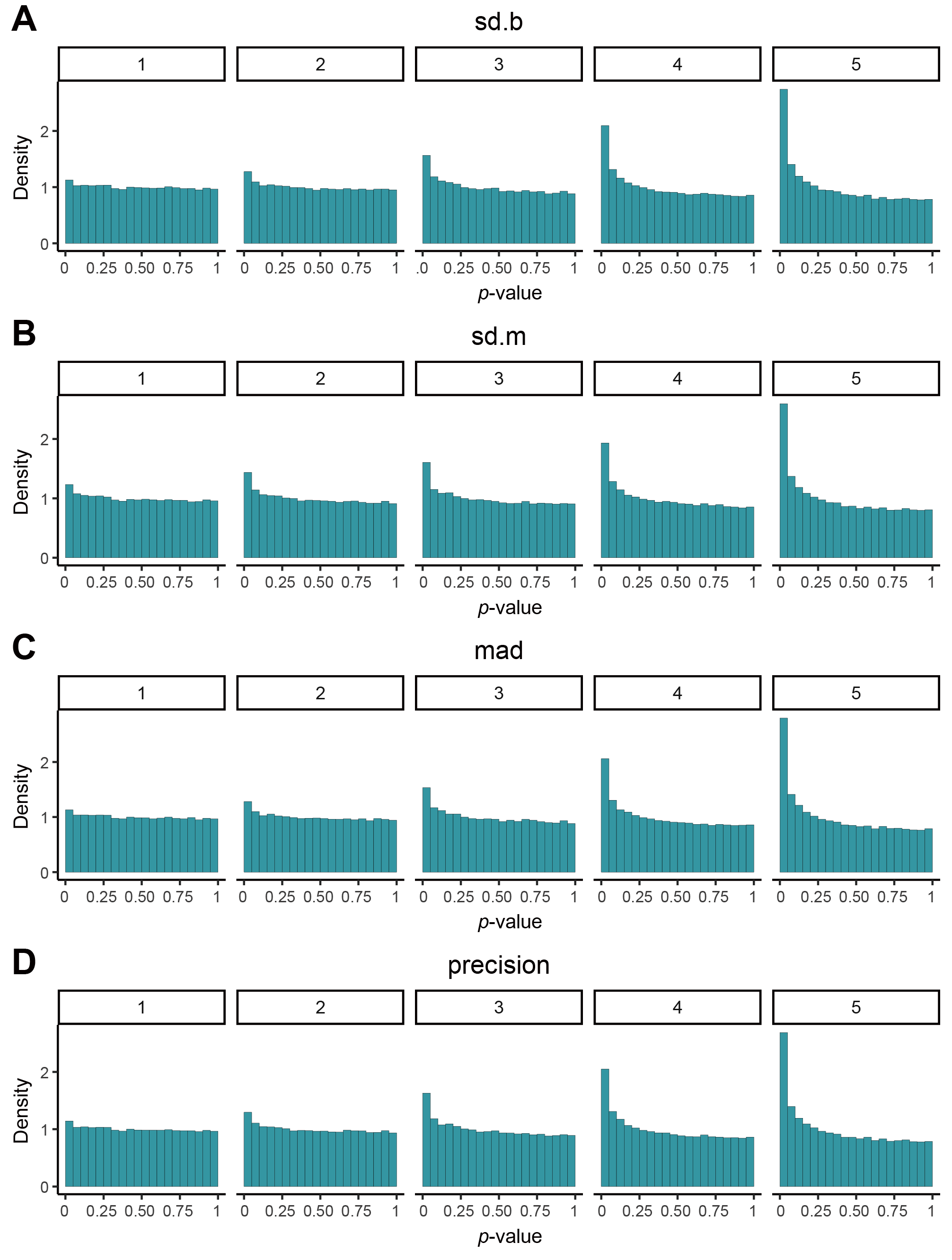

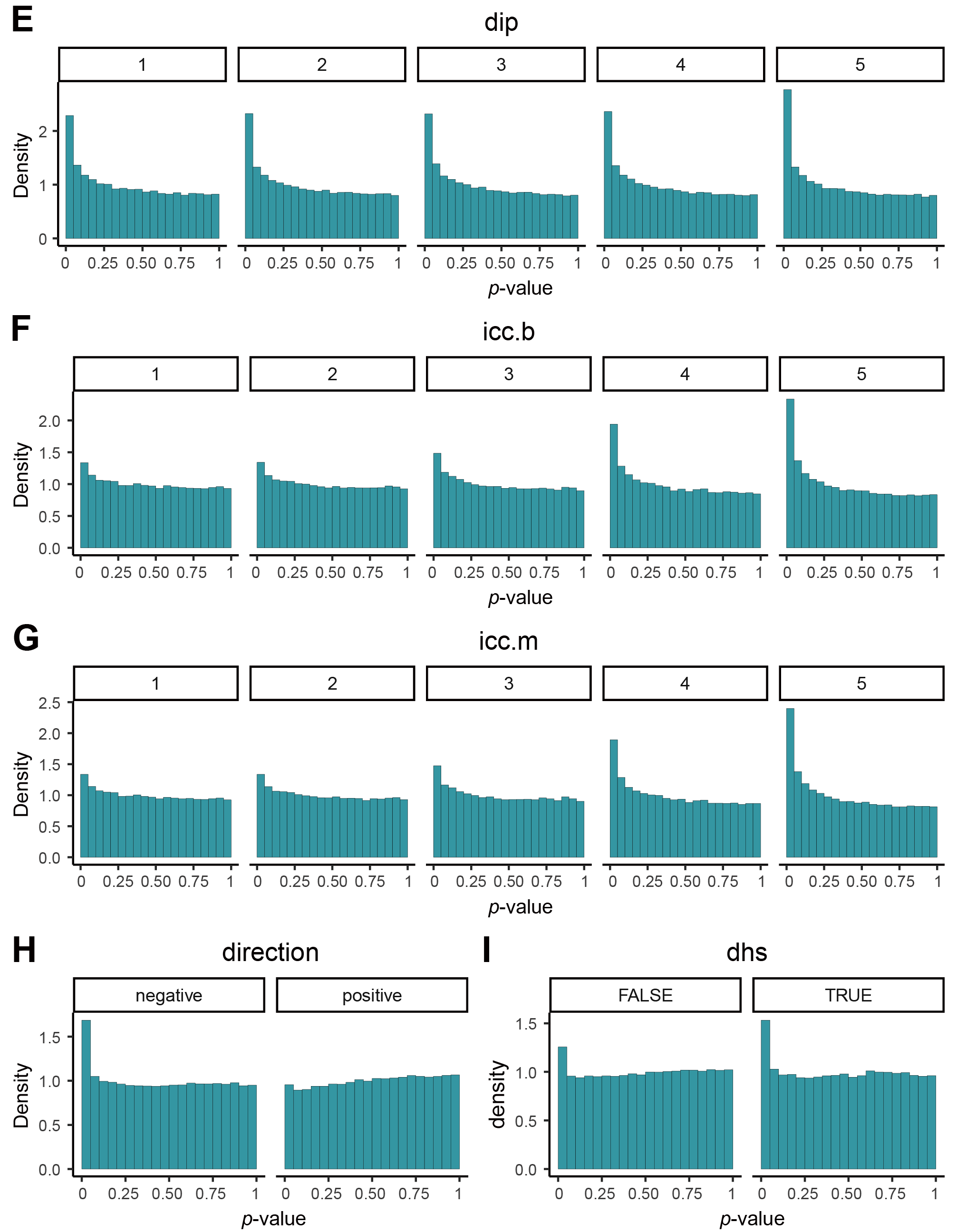

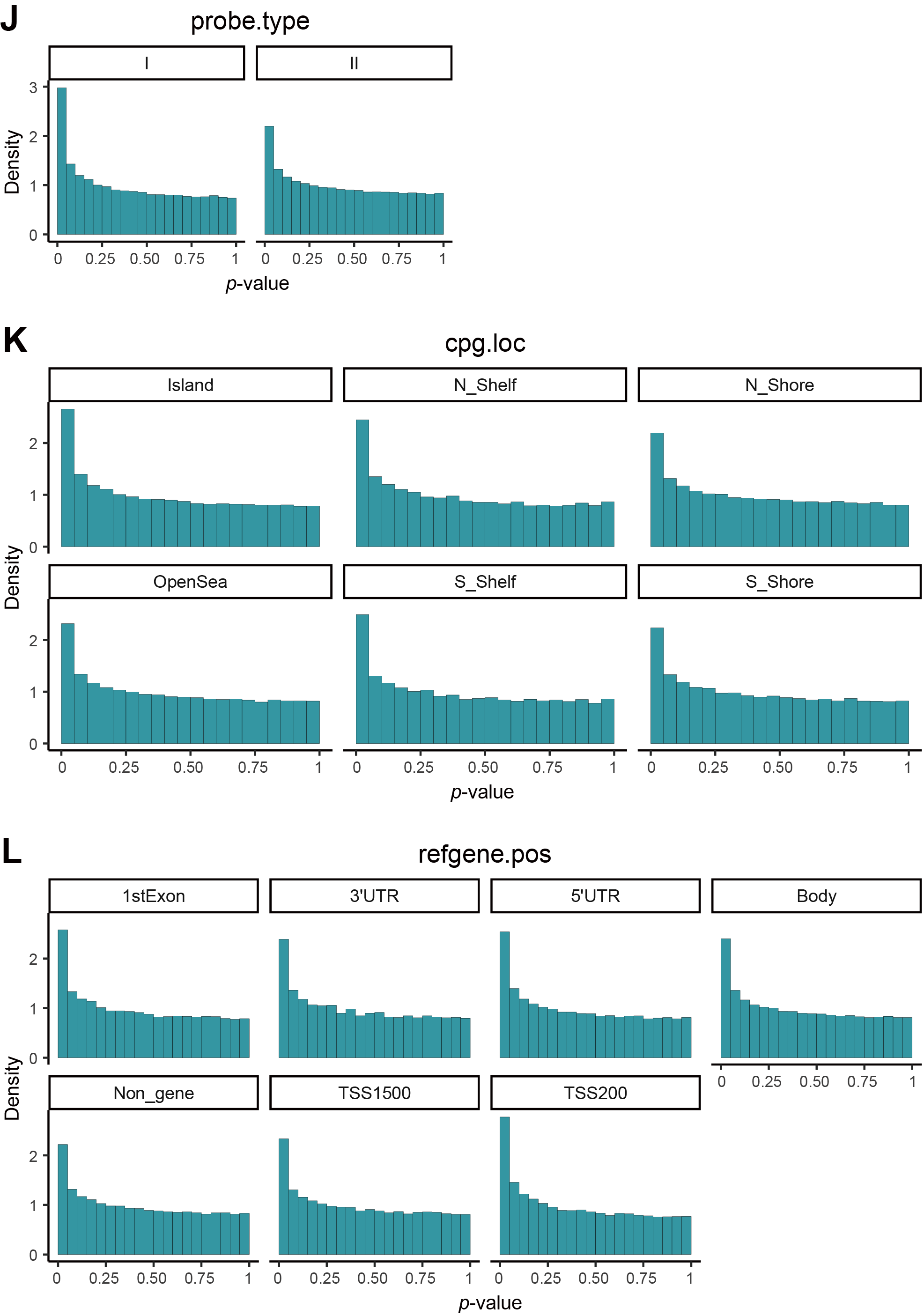


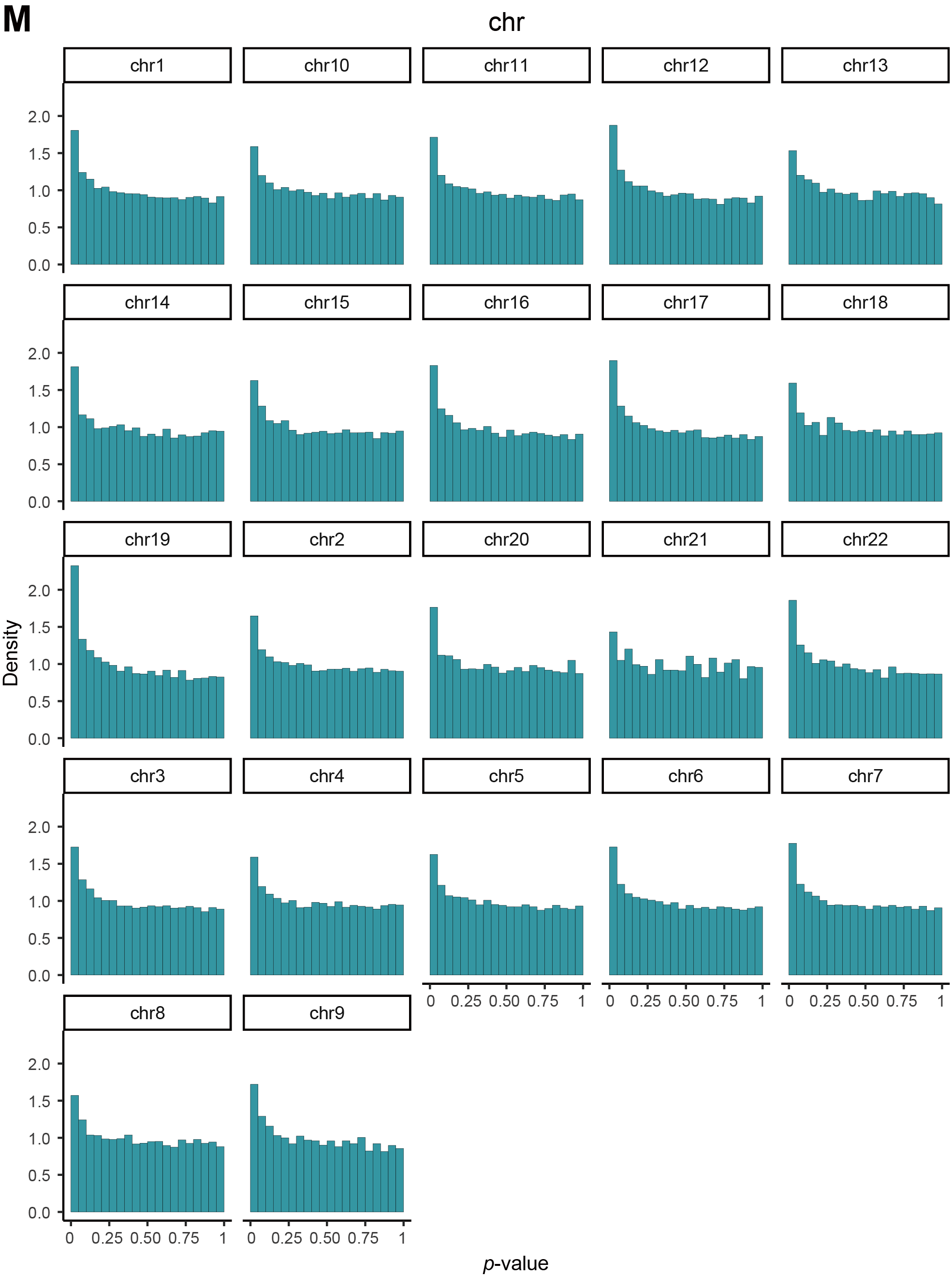


**Figure S4 Example histograms of *p*-values stratified by quintiles of different covariates.** Covariate "sd.b", "sd.m", "mad", "precision", "dip", "icc.b", "icc.m", "direction", "dhs", "probe.type", "cpg.loc", "refgene.pos", and "chr" are shown in A-M, respectively.

**
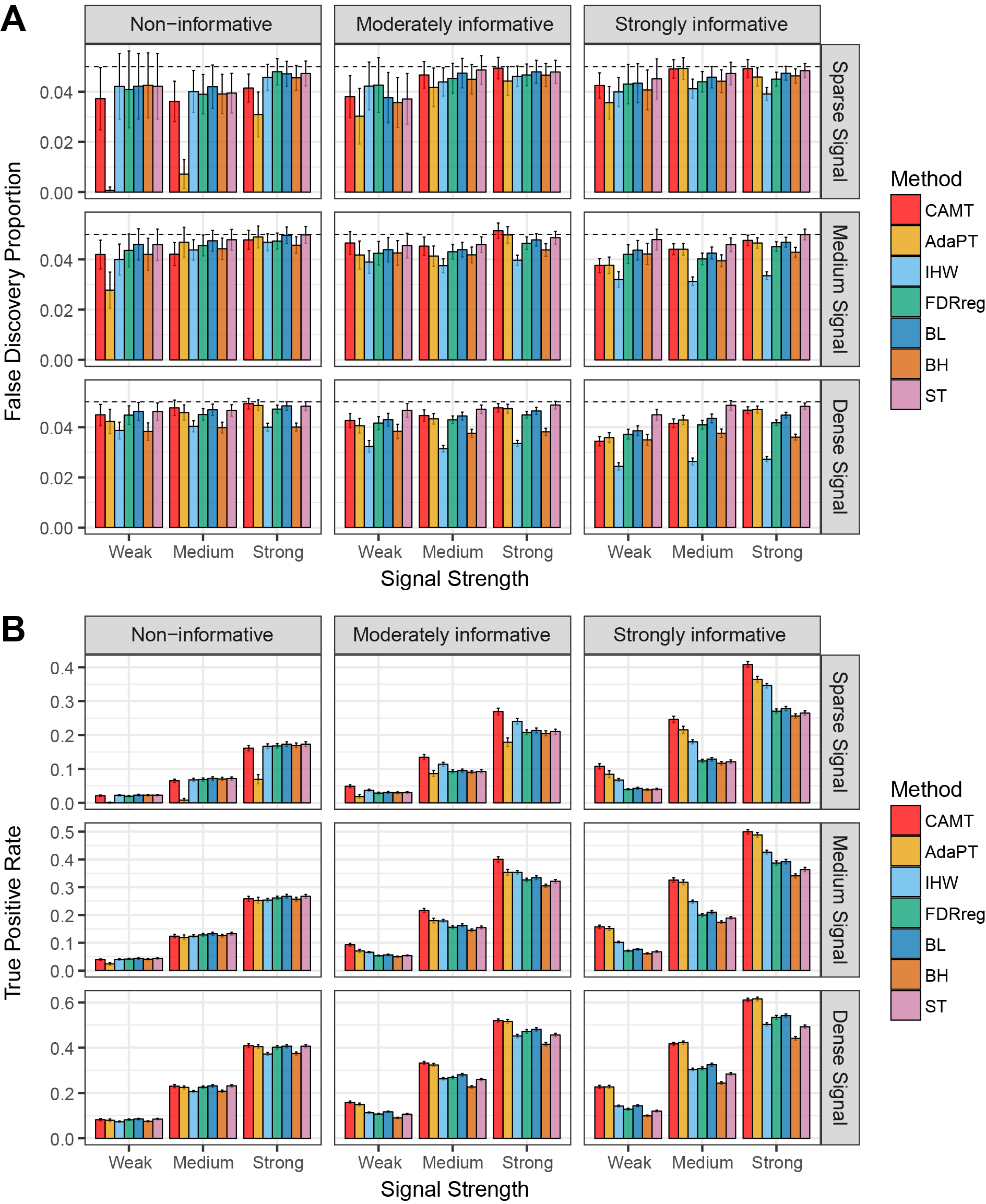
**

**Figure S5** **Performance evaluation of covariate adaptive FDR control methods on simulated data (correlated signals)**. Performance is evaluated based on FDR control (average false discovery proportion, panel A) and power (average true positive rate, panel B). The performance is compared under combinations of different level of covariate informativeness ("Non-informative", "Moderately informative" and "Strongly informative", columns), signal density ("Sparse signal", "Medium signal" and "Dense signal", rows) and signal strength ("Weak", "Medium", "Strong"). Error bar indicates the standard error across 100 repetitions, and the dashed line denotes the target FDR level (0.05).


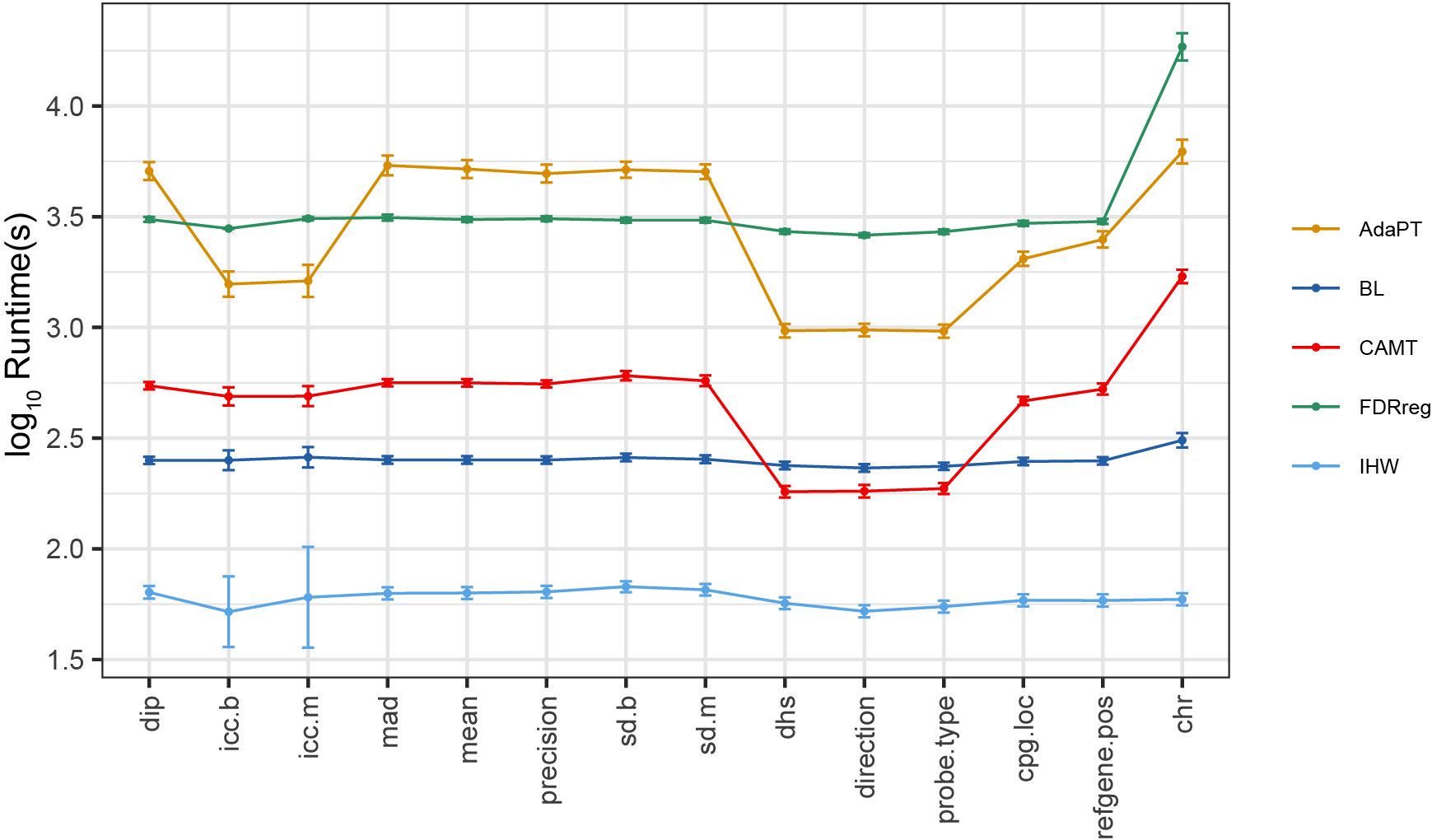


**Figure S6 Comparison of the run time (log10 scale) with different covariates**. The average run time was calculated over 61 EWAS datasets except for covariates "icc.b", " icc.m" and "direction", since they were only available for 3, 3 and 54 EWAS datasets, respectively. The error bar indicates 95% confidence interval.


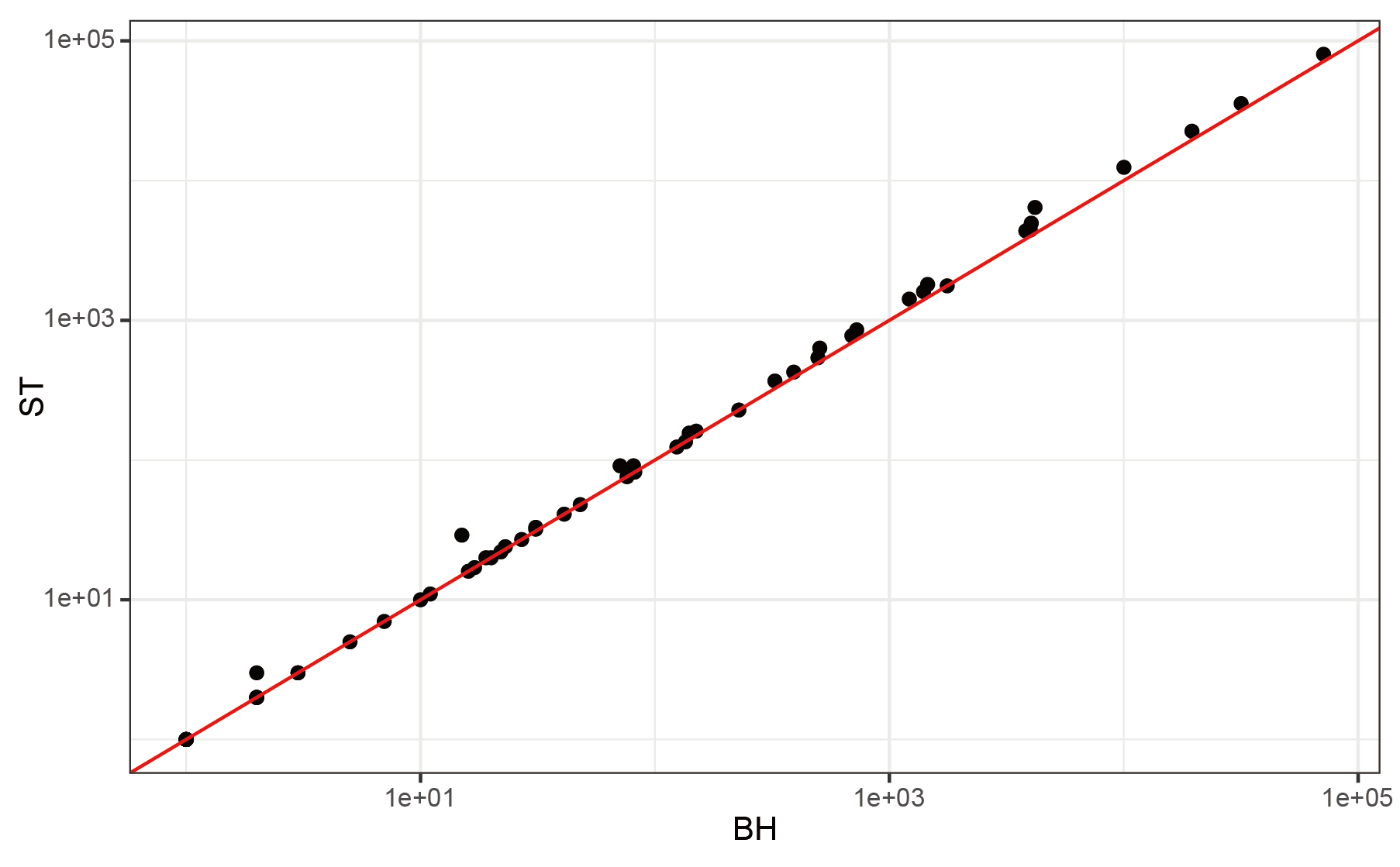


**Figure S7** **Comparison of the number of detected DMPs between BH and ST**. Axes are on the log10 scale, and the diagonal red line indicates y=x.


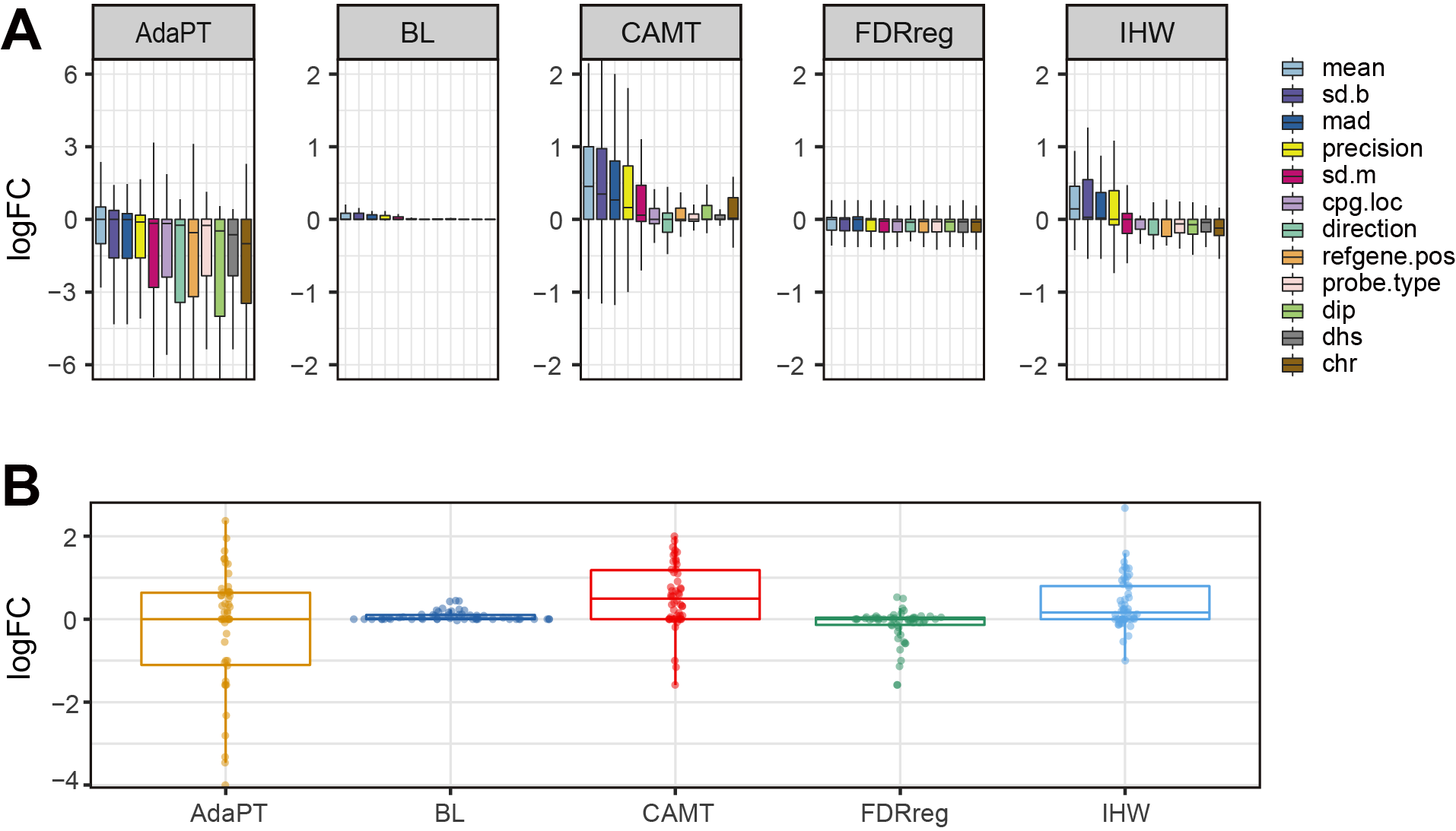


**Figure S8** **Comparison of detection power between different methods.** (A) Distribution of the log fold changes (logFCs, w.r.t. ST) in detected DMPs for different method-covariate combinations across the 61 EWAS datasets. (B) Distribution of the log fold changes in detected DMPs for different methods when their most informative covariate is used.


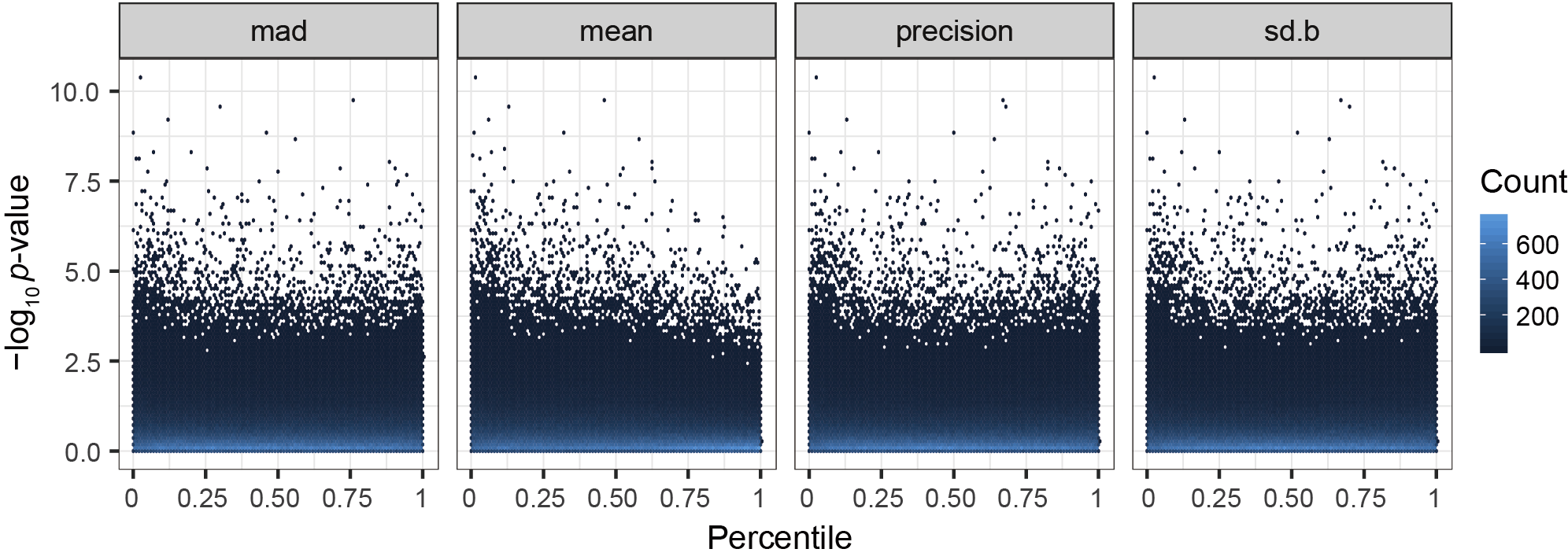


**Figure S9 An example where covariate "mean" is much more informative than the covariate measuring variance ("mad", "mean", "precision" and "sd.b")**


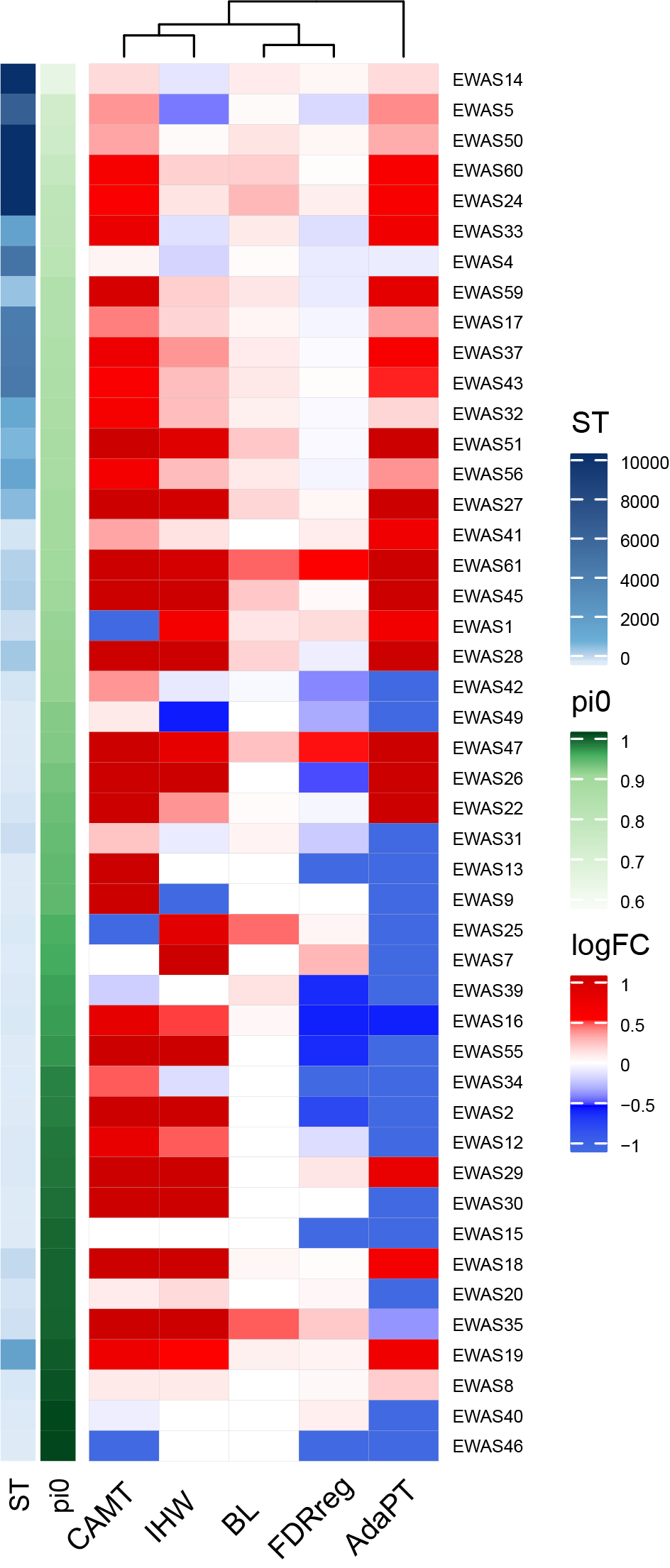


**Figure S10 Performance comparison of covariate-adaptive FDR control methods with the most informative covariate.** The detection power was computed as log2 fold change (logFC) to the ST method, all the numbers were added a pseudo-count of 1 to avoid 0s. For each EWAS dataset, the most informative covariate based on the omnibus test was used. The two left sidebars indicate the number of DMPs detected by ST with a target FDR level 5% and the estimated signal density (pi0), respectively. Fifteen out of 61 datasets are not shown here due to lack of significant informative covariates.


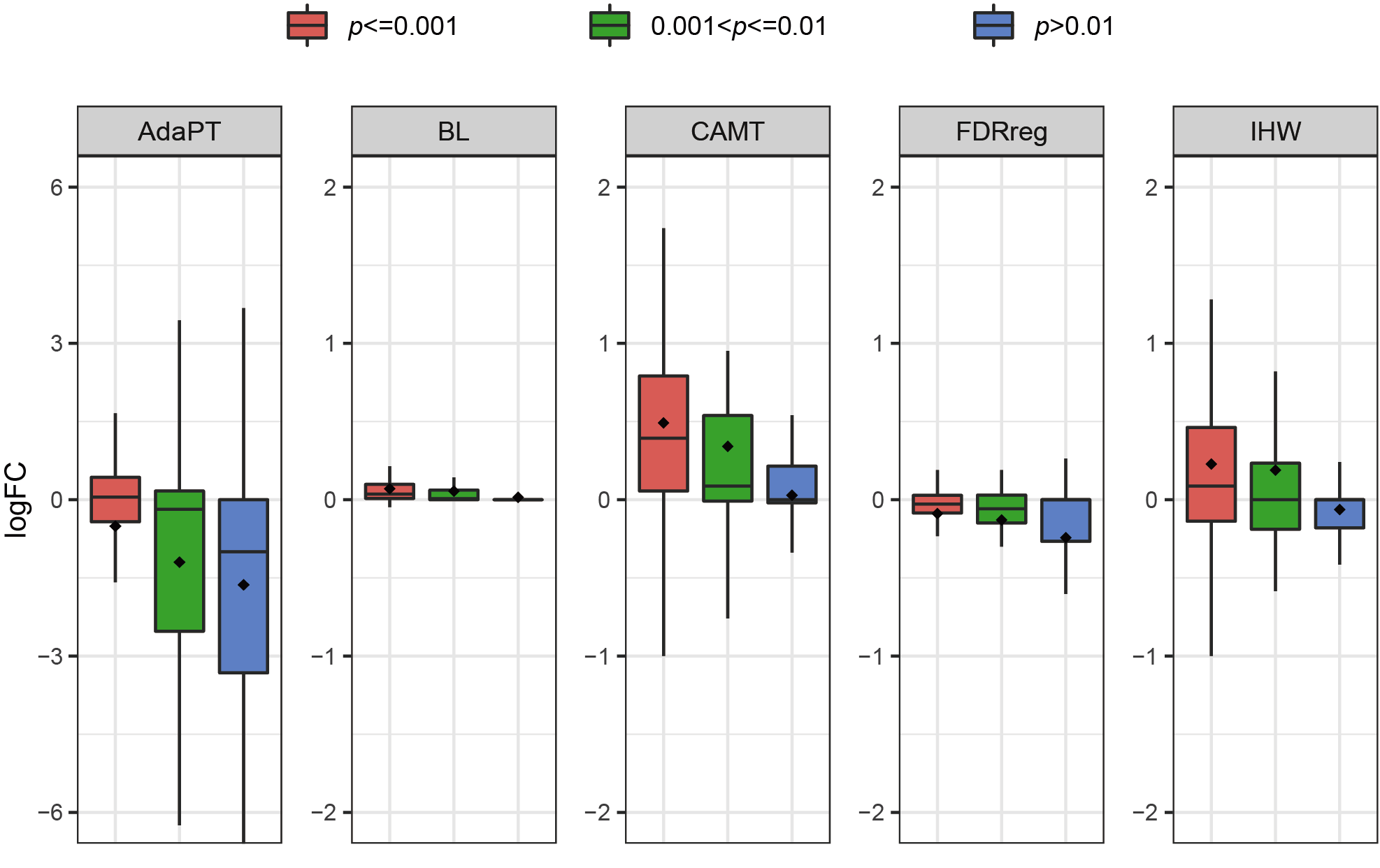


**Figure S11 Distribution of the log fold changes (logFCs, w.r.t. ST) in detected DMPs for different methods stratified by omnibus *p*-value level.** The diamond on each boxplot indicates the mean value of that group.

**
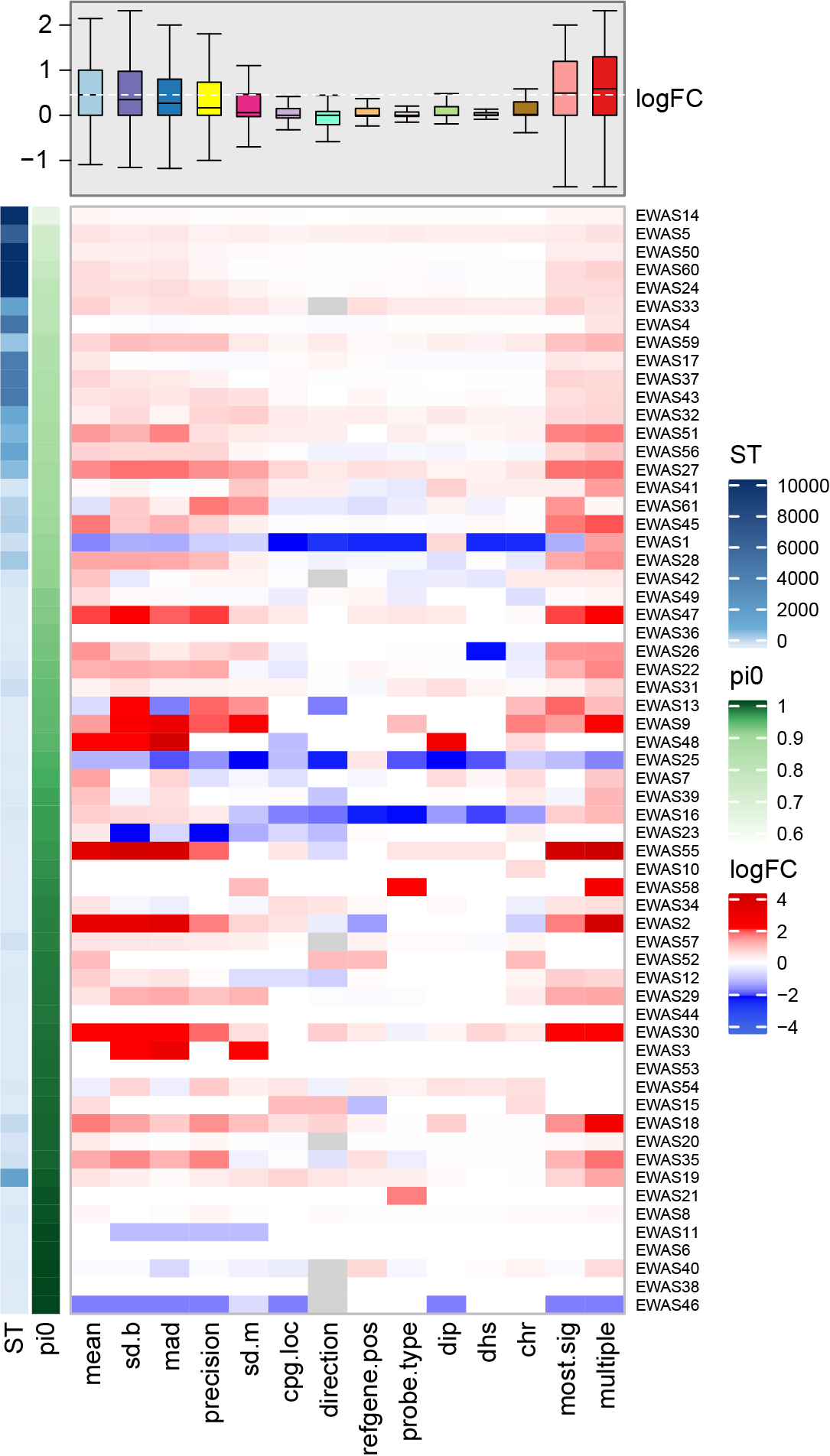
**

**Figure S12 Heatmap of detection power of CAMT with various covariates.** Detection power was computed as log2 fold change to ST method, and pseudo-count of 1 was added to all numbers. The last two columns "most.sig" and "multiple" represent the strategy using the most informative covariate and the combination of all informative covariates (omnibus test *p*-value < 0.05), respectively. The two left sidebars indicate the number of DMPs detected by ST with a target FDR level 5% and the estimated signal density (pi0), respectively. The top panel shows the boxplot of detection power in terms of log2 fold changes across datasets.


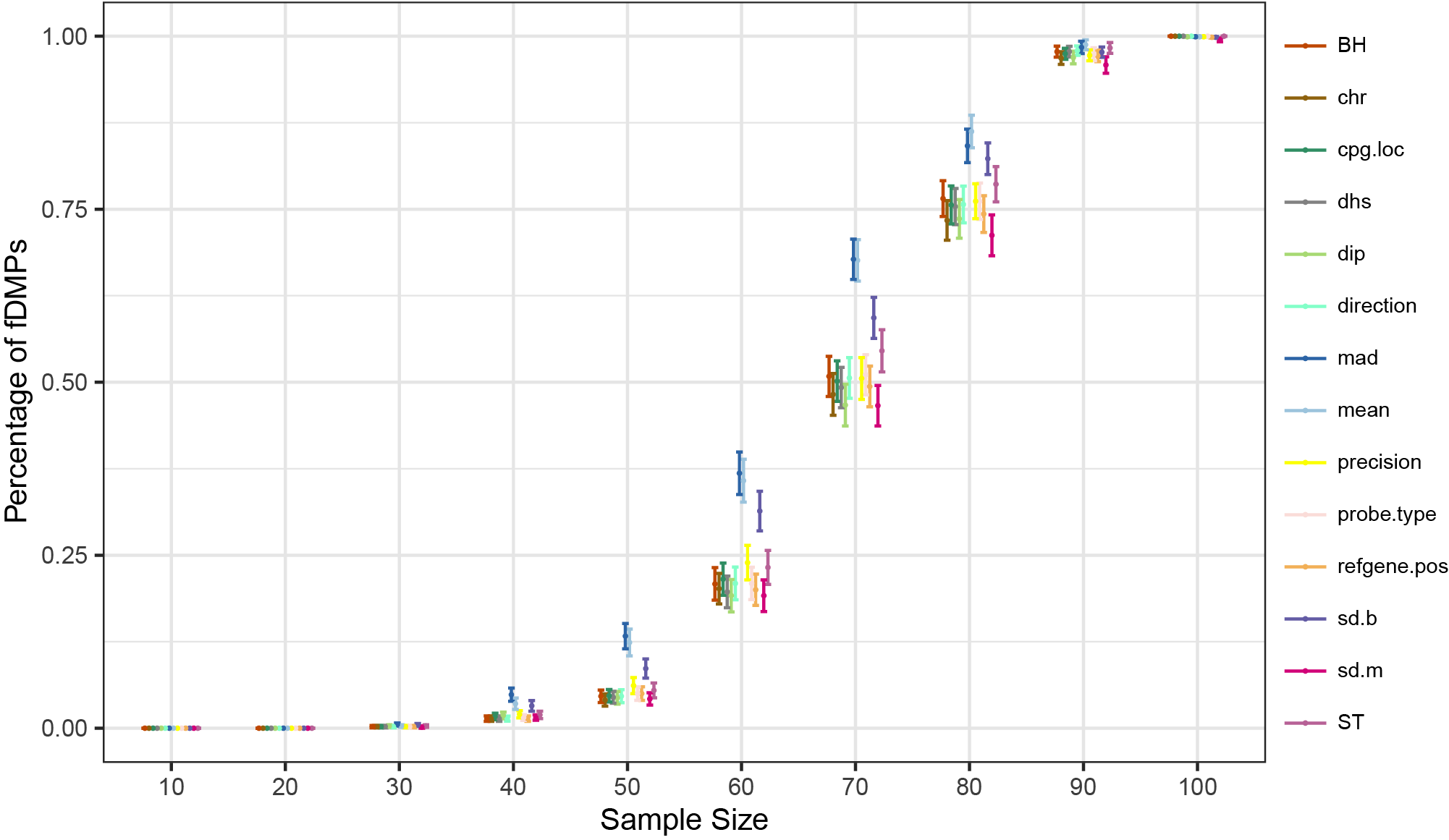


**Figure S13** **Covariate adaptive FDR control methods increase the detection power based on the down-sampling analysis.** The results are based on the IHW method. Y-axis shows the percentage of fDMPs (DMPs using the *full* dataset) recovered at sample size 10, 20, 30, 40, 50, 60, 70, 80, 90, and 100 for each group. Error bar indicates the standard error.


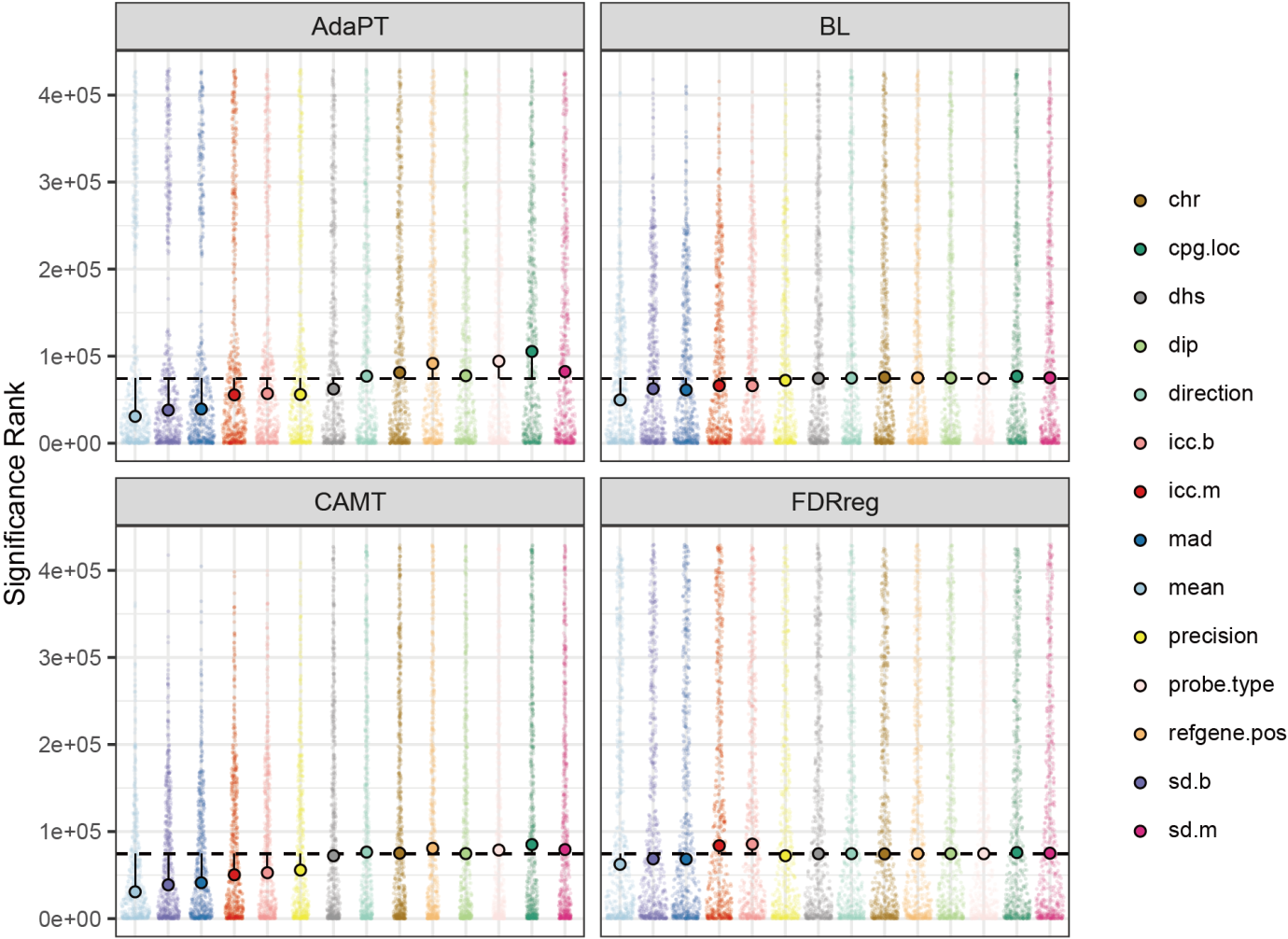


**Figure S14 Distribution of the significance rank of the gold standard age-associated DMPs (aDMPs) for different covariates using AdaPT, BL, CAMT, and FDRreg**. A lower rank indicates a more significant result. The circle represents the median rank of aDMPs for the specific covariate. The dashed line indicates the median rank of aDMPs for the ST method.


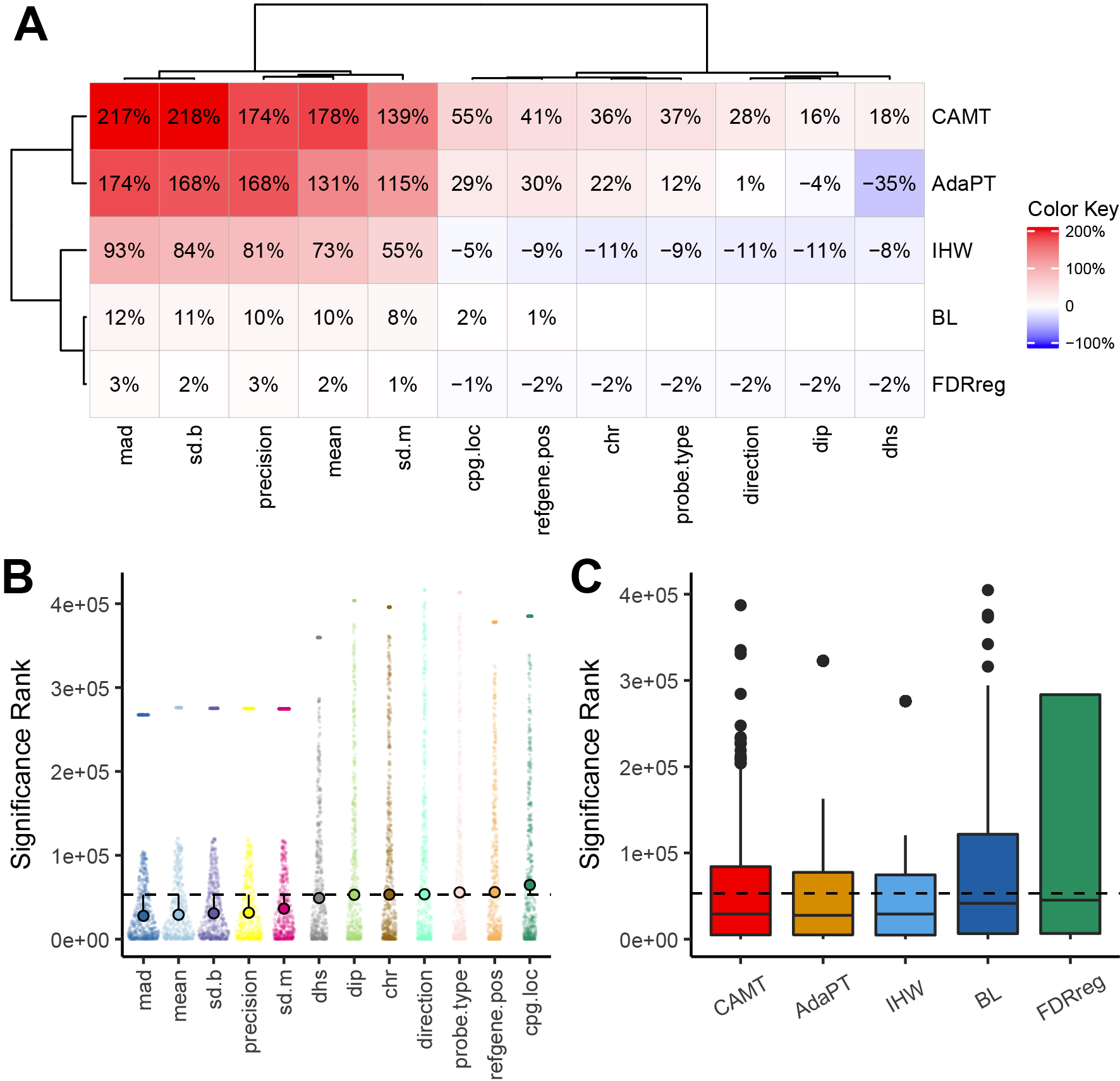


**Figure S15** **Covariate adaptive FDR control methods increase the power to detect age-associated DMPs**. A different EWAS dataset (EWAS27) was used. (A) Percent changes in detected DMPs from the ST method for different method and covariate combinations. Changes with more than 1% are displayed in corresponding cells. (B) Distribution of the significance rank of the gold standard age-associated DMPs (aDMPs) for different covariates using the IHW method. The circle represents the median rank for the specific covariate. A lower rank indicates a more significant result. The dashed line indicates the median rank for the ST method. (C) Distribution of significance rank of the aDMPs for different methods using the methylation "mean" as the covariate.


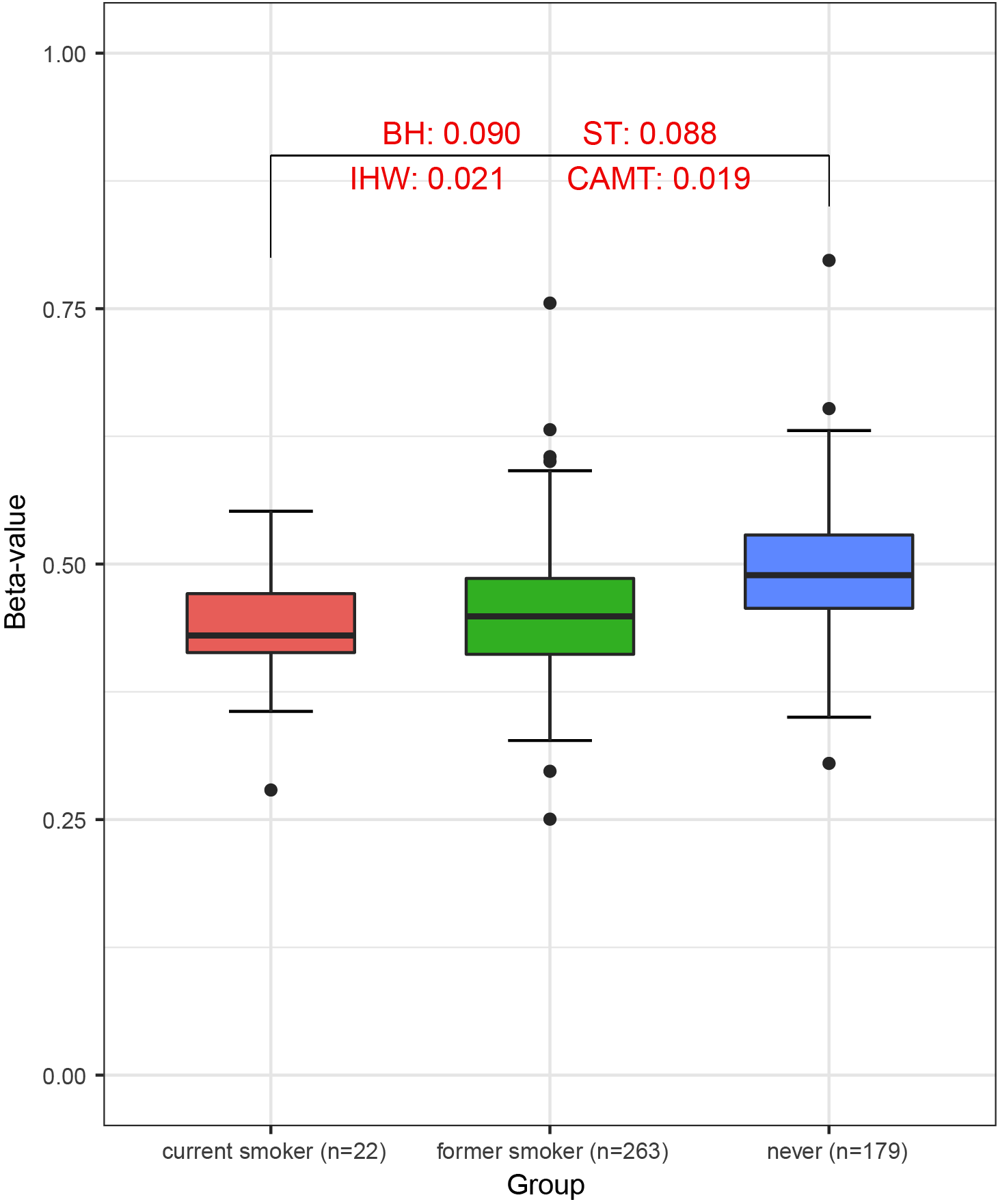


**Figure S16** **Comparison of the methylation level of cg18092474 among former smokers, current smokers and never smokers**. FDR adjusted *p*-values are shown for method BH, ST, IHW and CAMT with the covariate "mean".


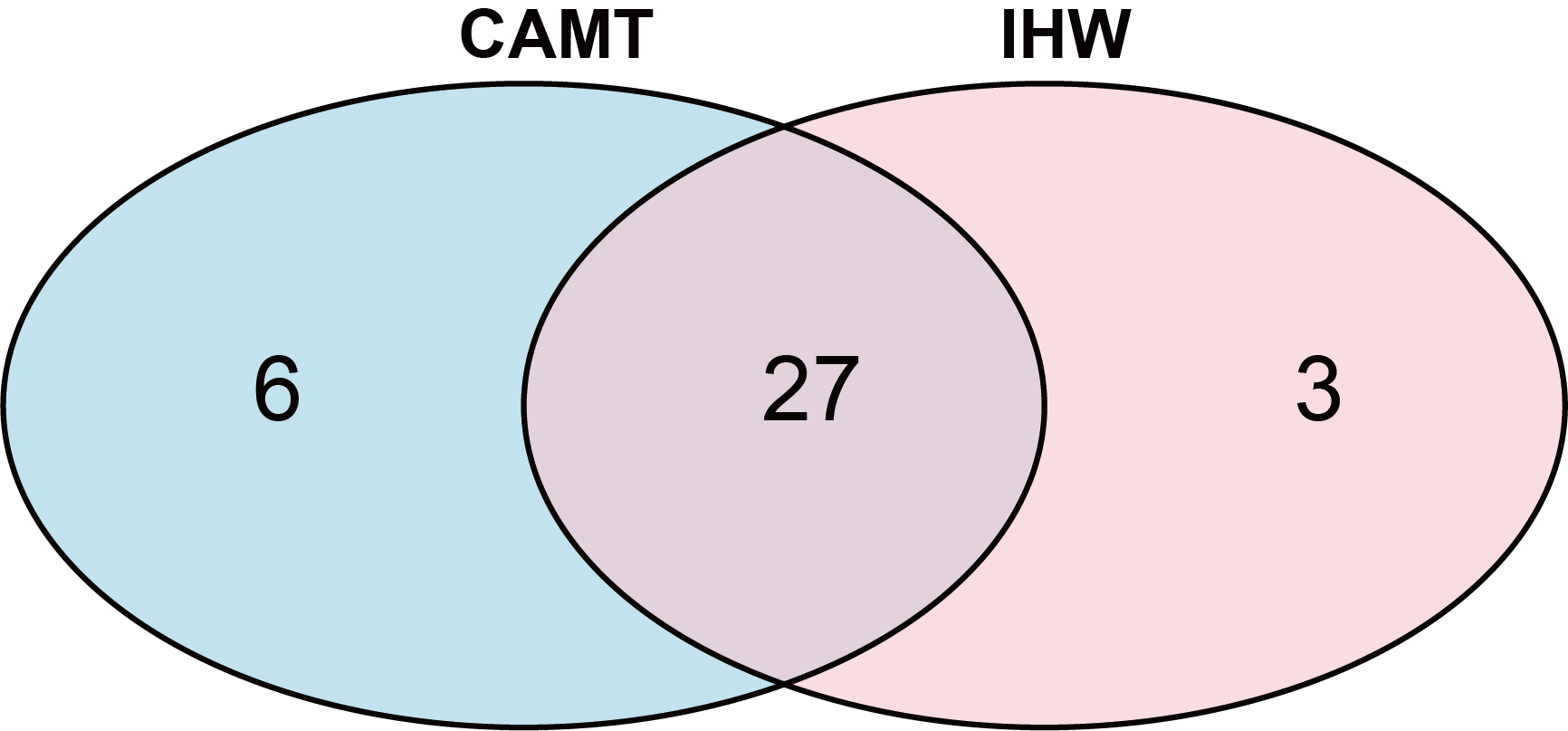


**Figure S17** **Overlap of DMPs between IHW and CAMT**. The results are based on pooling DMPs from all covariates.


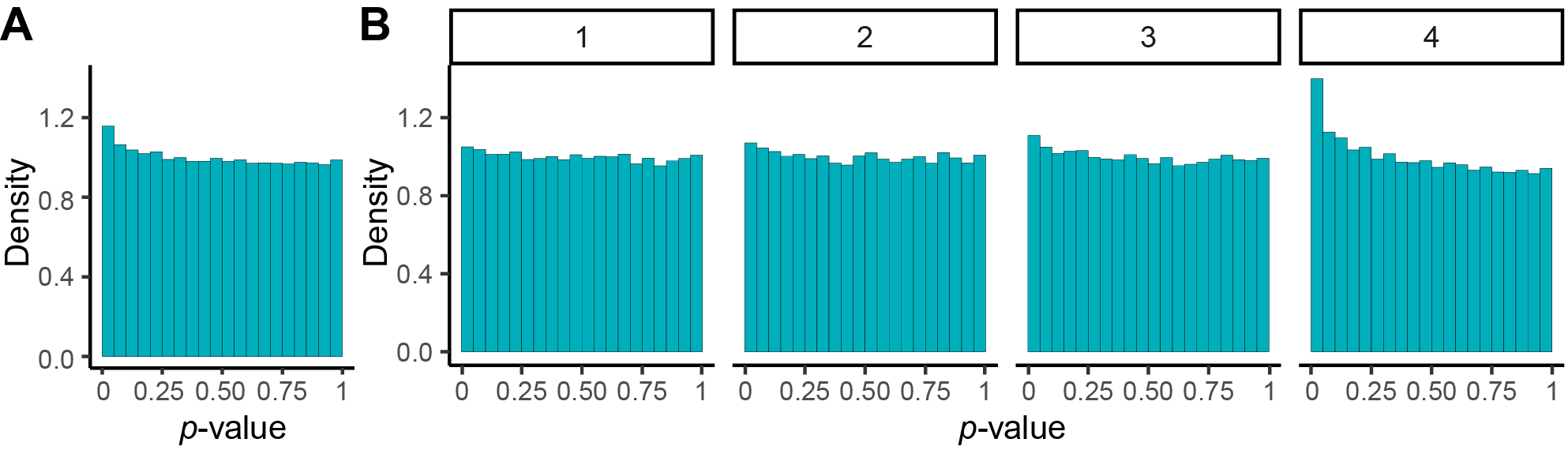


**Figure S18 Histograms of the association *p*-values for EWAS29.** (A) Histogram of all *p*-values from EWAS29. (B) Histograms of the *p*-values from EWAS29 stratified by the *z*-scores (transformed *p*-values) from EWAS28.


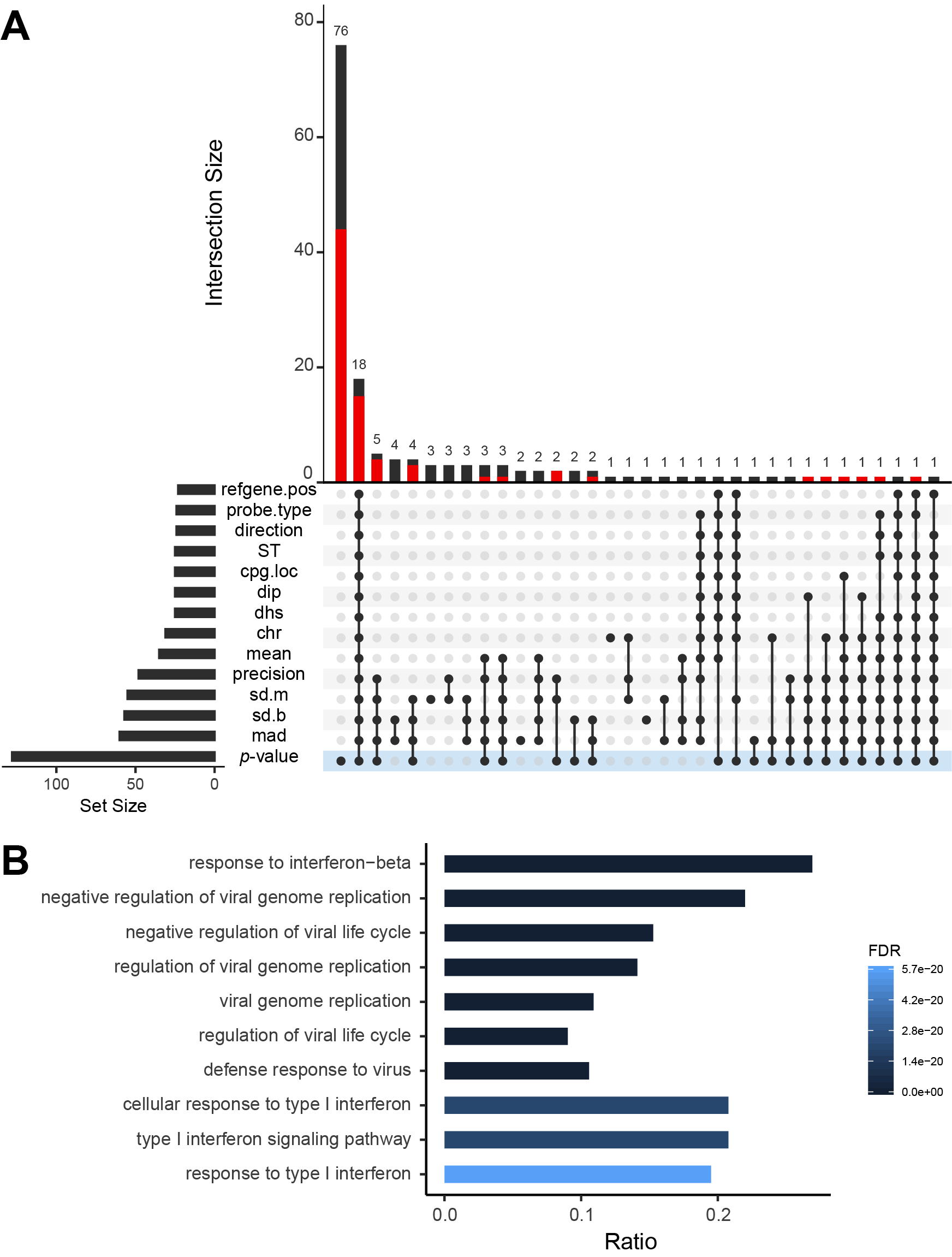


**Figure S19** **The use of the association *p*-value from a related study as a covariate.** The *p*-values from an EWAS of Systemic Lupus Erythematosus (SLE) using CD19+ B-cells (EWAS28) were used as the covariate to adjust the *p*-values for another EWAS of SLE using CD4+ T cells (EWAS29). (A) Intersections in detected DMPs for the CAMT method using different covariates. The connected dots indicate the intersected covariates. Left sidebar indicates the number of DMPs detected by the IHW method using different covariates. As a comparison, the ST method is also included. The top sidebar indicates the intersection size and the red part indicates the number of DMPs detected by EWAS28. (B) Top 10 GO terms from enrichment analysis based on the CAMT method using the *p*-value from a related study as the covariate. The x-axis indicates the percentage of DMPs in the enriched biological process (out of the total DMPs). And GO terms from top to bottom are arranged in descending order (top 1 to top 10).


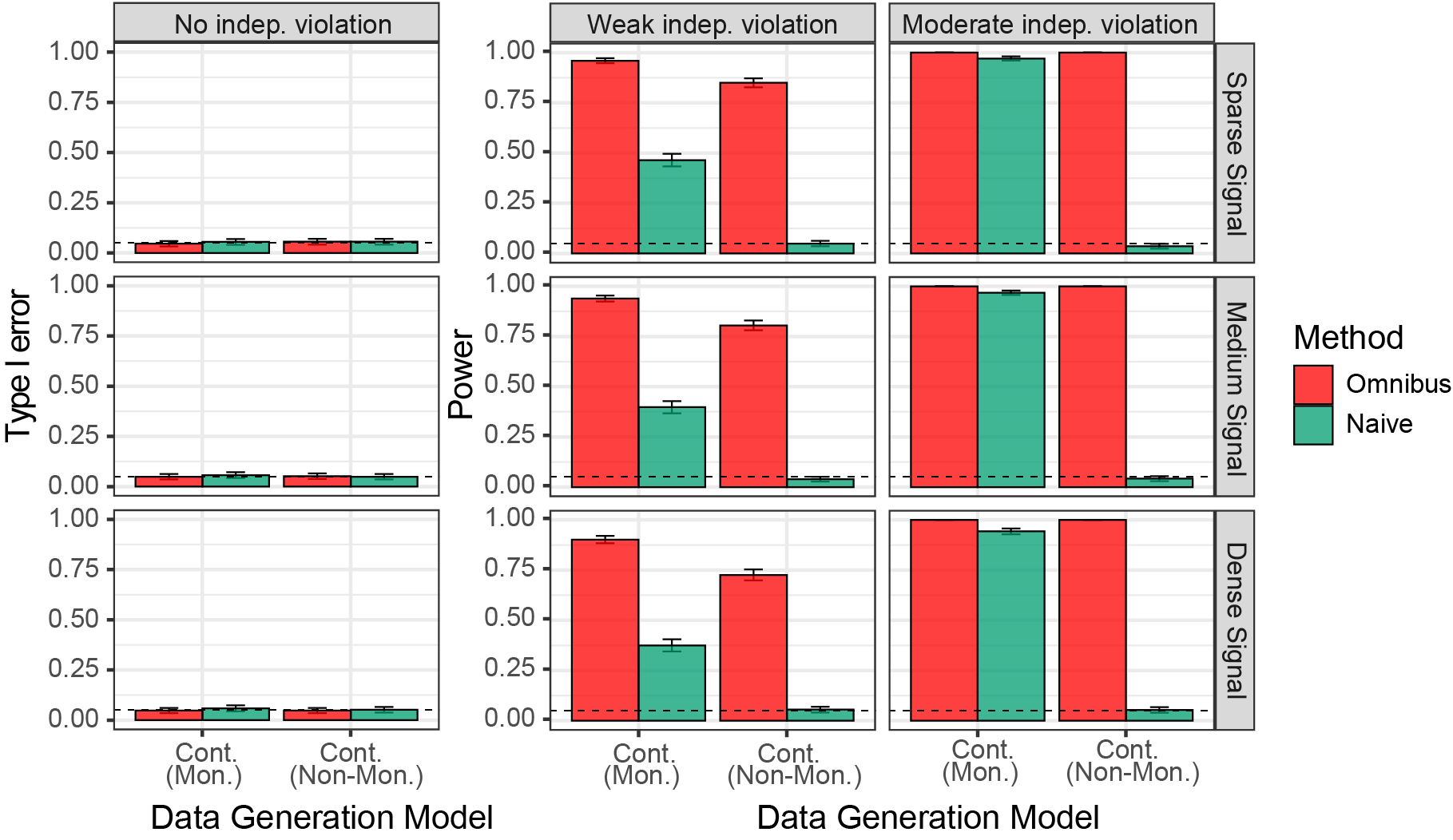


**Figure S20 Type I error and power to detect the violation of the “independence under the null” assumption based on a variant of the omnibus test.** The simulation is similar to that described in the method section “Simulations for studying the performance of the omnibus test.” The differences are: (1) we do not simulate dependency under the alternative (c=0), (2) we simulate dependency under the null (violation of the assumption) and (3) we only investigate a continuous covariate. To simulate weak and moderate violation of the assumption, we randomly draw 5% and 10% of the null *p*-values and order them by the corresponding values of the covariate ($x_{i}$ or $x_{i}^{2}$) to create dependency. "Cont.(Mon.)" and "Cont.(Non-Mon)" represent continuous covariates with a monotonic dependence, and continuous covariates with a non-monotonic dependence, respectively. Naïve test refers to Spearman's rank correlation test. The nominal level for type I error is 0.05 (dashed line).
