# Supplementary material for: Leveraging biological and statistical covariates improves the detection power in epigenome-wide association testing
Source: Genome Biol. 2020 Apr 6;21:88. doi: 10.1186/s13059-020-02001-7 (PMC7132874; doi:10.1186/s13059-020-02001-7)
Supplement: Supplementary file 7 — Review history. [file 13059_2020_2001_MOESM7_ESM.docx]

Review History

**First round of review**

**Reviewer 1**

**Comments to author:**

This is a well-done paper in which the authors used publicly available DNA methylation 450K data to evaluate various covariate-adaptive FDR methods.  The authors proposed an omnibus test to assess the dependency between association p-values and each covariate under EWAS settings.  The evaluations were thorough, and the results were presented clearly.
I only have some minor comments:
1)     The authors have summarized the results well from different FDR methods and covariates, but did not discuss fully why some covariates performed better than others, except for the mean.  It is not surprising to see that the statistical covariates (mean, sd, mad, etc.) are more informative than the biological/technical covariates, as the former are (or related to) important factors to statistical power.  However, as described in the text, the EWASs were conducted on M-values, but sd.b improved power more than sd.m. Any possible reasons behind this observation?
2)     The authors proposed an omnibus test to evaluate the covariates.  Although it performs well in the simulation studies, the authors did not emphasize the use of this test when using real EWAS datasets.
3)     A very minor comment: it may be worthwhile to clarify in the Methods section that the 61 EWASs were for different outcomes.  There are a lot of results, and I was somewhat lost when reading to the part of real data analysis.

**Reviewer 2**

**Comments to author:**

Review of "Leveraging biological and statistical covariates improves the detection power in epigenome-wide association testing" by Huang, Bai, and Cui et al. This manuscript proposes an omnibus testing procedure for whether or not a covariate is informative for use with covariate-adaptive FDR control methods. It also provides an extensive evaluation of various FDR control methods & informative covariates under a very wide range of EWAS datasets. The authors should be commended for producing a well-written article with clearly presented results. The proposed omnibus test for covariate informativeness is potentially extremely useful to anyone applying covariate-adapted FDR methods. The extensive case studies are of high interest to those in the EWAS field, as well as those interested in comparing performance of covariate-adaptive FDR methods. Below, I provide a few major and minor suggestions to strengthen the results of the paper.

Major comments:
1.     The simulations to evaluate type I error and power of the proposed omnibus test are a great start, but another equally important evaluation is how the test performs when the covariate is informative but not independent under the null. It is my understanding that none of the tested simulation scenarios exhibit this property. If the test results in false detection of these covariates, then it this is a limitation of the test that the stratified histograms still need to be checked, reducing the utility of the test.
2.     I think that the results of Figures S9-S10 show one of the most useful results of the manuscript (the utility of the omnibus test in achieving performance gain with various methods). It also leads to questions about how to use these results to better interpret the results in Figure 3. It would be interesting to see a subset version of Figure three, which only gives results for a given dataset/covariate combination if the omnibus test passed a given threshold (missing otherwise). Or just gives the results for the most significant covariate per dataset (collapsed into 1 column).
3.     The analyses using a p-value from a related disease/phenotype as a covariate (Figure 6): if the sets of p-values are too similar, the independence under the null assumption has potential to be violated. Has this been checked in this case?
4.     A note on possible interpretation of the reason AdaPT suffered from a great power loss when covariate is uninformative: Please see the section "Including an uninformative covariate is not harmful" in [1]. It seems that there is a simple remedy that can be invoked by the user when applying the AdaPT method, but the option is not made default nor mentioned in AdaPT's documentation despite being suggested by the authors of [1], which states that the AdaPT authors "responded positively to the recommendation of documenting this procedure in future releases of the package." A quick check reveals that this has not been done as of December 2019, and although I don't think it should not be the responsibility of the authors of this manuscript under review to implement this extra procedure on behalf of the AdaPT developers, it would be prudent to mention this issue since the findings are consistent with [1].

Minor comments:

1.     Figure 1A: it is confusing to have the y-axis labeled as both Type I error and Power. Although it is clarified in the legend, I think interpretation of the figure would be easier if it was broken into two (one for the first column / Type I error and another for the second and third / power).
2.     Figure S6: are these results averaged over all 61 datasets? This should be clarified in legend
3.     The interpretation of the results of Figure 3 in the text does not point out how visually the CAMT method seems to perform the best. Indeed, there are a few cases where the performance is worse than ST, but this is to a lesser degree than any of the other methods.

References:
[1] Korthauer K, Kimes PK, Duvallet C, Reyes A, Subramanian A, Teng M, Shukla C, Alm EJ, Hicks SC. A practical guide to methods controlling false discoveries in computational biology. Genome biology. 2019 Dec;20(1):118.

**Authors Response**

**Point-by-point responses to the reviewers’ comments:**

    Reviewer #1: This is a well-done paper in which the authors used publicly available DNA methylation 450K data to evaluate various covariate-adaptive FDR methods.  The authors proposed an omnibus test to assess the dependency between association p-values and each covariate under EWAS settings. The evaluations were thorough, and the results were presented clearly.

    I only have some minor comments:

1) The authors have summarized the results well from different FDR methods and covariates, but did not discuss fully why some covariates performed better than others, except for the mean.  It is not surprising to see that the statistical covariates (mean, sd, mad, etc.) are more informative than the biological/technical covariates, as the former are (or related to) important factors to statistical power.  However, as described in the text, the EWASs were conducted on M-values, but sd.b improved power more than sd.m. Any possible reasons behind this observation?

**Answer and response:** *We greatly appreciate the kind suggestion of the reviewer. We now expanded the discussion about the covariates, which we also copied below:*

“For EWAS data, we investigated 14 potential useful covariates. We found that statistical covariates are more informative than biological/technical covariates, as the former are more directly related to statistical power. In addition, statistical covariates could also be informative of the prior null probability.  For example, the variance of the feature is considered to be a good indicator of the null probability [[40](https://docs.google.com/document/d/1L7tcxoSiMXCFDsCbk8JXrOH5rh6xwQjk/edit#heading=h.30j0zll)] since an invariant feature is less likely to be differential. Variance-based filters have been frequently used to exclude invariant genomic features to reduce multiple testing burden [[68](https://docs.google.com/document/d/1L7tcxoSiMXCFDsCbk8JXrOH5rh6xwQjk/edit#heading=h.1fob9te)] and increase statistical power. One advantage of using covariate-adaptive methods is that filtering is no longer necessary, and the method could automatically down-weight these less likely features according to their variances. Interestingly, "sd.b" performed better than "sd.m" on the real data even though the association tests were performed on the M-values. This could be explained by the fact that "sd.b" as the standard deviation on the Beta-value might be a more biologically relevant measurement of methylation variability and is more informative of the null probability.  The strong performance of the covariate "mean" is somewhat unexpected and may be explained by two reasons, which are related to the prior null probability and statistical power, respectively. First, DMPs are enriched in certain regions such as the promoters [[30](https://docs.google.com/document/d/1L7tcxoSiMXCFDsCbk8JXrOH5rh6xwQjk/edit#heading=h.3znysh7), [31](https://docs.google.com/document/d/1L7tcxoSiMXCFDsCbk8JXrOH5rh6xwQjk/edit#heading=h.2et92p0)], where the methylation levels are similar – either low or high (Additional file 2: Figure S3A).  Second, the statistical power to detect DMPs in the middle of the methylation spectrum is higher than those in the two ends, where the methylation changes are constrained (Additional file 2: Figure S3B).  The covariate "dip" was the least informative among all statistical covariates since the majority of CpGs, including those DMPs, have unimodal distributions and the dip statistic is not very informative. Compared to statistical covariates, external covariates were less universal than statistical covariates, and they were informative only for specific datasets. For instance, the covariate "cpg.loc" was very powerful for EWAS19 since DMPs in pancreatic ductal adenocarcinoma were enriched in CpG islands [[20](https://docs.google.com/document/d/1L7tcxoSiMXCFDsCbk8JXrOH5rh6xwQjk/edit#heading=h.tyjcwt)].”

2)	The authors proposed an omnibus test to evaluate the covariates. Although it performs well in the simulation studies, the authors did not emphasize the use of this test when using real EWAS datasets.

**Answer and response:** *Thanks for raising this important point. We now generated one more supplementary figure (Additional file 2: Figure S12) to illustrate the potential use of the omnibus test in real EWAS data using CAMT as an example.  The figure presented the results of CAMT only (part of Figure 3), adding two potential strategies to use the omnibus test results (the last two columns).  The first strategy (‘most.sig’) used the most significant covariate and the second strategy (‘multiple’) combined all the significant covariates (omnibus p < 0.05). We could see both approaches produced very robust results, the power improvement (logFC, top panel) were similar to or slightly better than the best covariate ‘mean’. We revised the text, which we also copied below:*

“Besides using the most informative covariate, combining all informative covariates is also possible for methods that accommodate multiple covariates such as CAMT.  We tried both strategies using CAMT. We could see that both approaches achieved quite robust results (Additional file 2: Figure S12), the power improvement (top panel) were similar to or slightly better than the best covariate "mean".”

    3)	A very minor comment: it may be worthwhile to clarify in the Methods section that the 61 EWASs were for different outcomes.  There are a lot of results, and I was somewhat lost when reading to the part of real data analysis.

**Answer and response:** *Thanks. We have made it clear in the text.*

    Reviewer #2: Review of "Leveraging biological and statistical covariates improves the detection power in epigenome-wide association testing" by Huang, Bai, and Cui et al. This manuscript proposes an omnibus testing procedure for whether or not a covariate is informative for use with covariate-adaptive FDR control methods. It also provides an extensive evaluation of various FDR control methods & informative covariates under a very wide range of EWAS datasets. The authors should be commended for producing a well-written article with clearly presented results. The proposed omnibus test for covariate informativeness is potentially extremely useful to anyone applying covariate-adapted FDR methods. The extensive case studies are of high interest to those in the EWAS field, as well as those interested in comparing performance of covariate-adaptive FDR methods. Below, I provide a few major and minor suggestions to strengthen the results of the paper.

Major comments:

    1.	The simulations to evaluate type I error and power of the proposed omnibus test are a great start, but another equally important evaluation is how the test performs when the covariate is informative but not independent under the null. It is my understanding that none of the tested simulation scenarios exhibit this property. If the test results in false detection of these covariates, then it this is a limitation of the test that the stratified histograms still need to be checked, reducing the utility of the test.

**Answer and response:** *Thanks for raising this insightful and practical point. Although the proposed omnibus test focuses on the association with the covariate in the small p-value region, it may also give false detection of these covariates that are strongly dependent of the p-value under the null. However, a variant of the omnibus test could be used to test the independence assumption under the null.  Specifically, we conduct the omnibus test using only the large p-values (p-value > 0.5, considered to be dominantly null p-values) and tailor the p-value cutoff points (***p(i)** 0.1, 0.2, 0.4, 0.6, 0.8, 0.9*-quantile of the p-values) to be more equally spaced. We show by additional simulations (see Additional file 2: Figure S20, simulation details in the legend) that this test is very powerful and can detect a moderate deviation from the assumption.  Therefore, in practice, we could conduct both types of omnibus test simultaneously, and select covariates that are significant in the first test (i.e., overall dependency between p-value and covariate) and not significant in the second test (i.e., the dependency is not due to violation of the independence assumption under the null).*

*We added a paragraph in the discussion to address this issue, which we also copied below:*

“The validity of these covariate-adaptive methods depends on the assumption that the p-value is independent of the covariate under the null.  This assumption is usually assessed empirically by stratified histograms: for large p-values (e.g., p-values ≥ 0.5), the distribution should appear uniform across strata. A variant of the omnibus test could be used to formally assess the assumption of independence under the null.  Specifically, the omnibus test can be focused on the large p-values (p-value > 0.5, considered to be dominantly null p-values) and tailor the p-value cutoff points to be more equally spaced (e.g., p(i) 0.1, 0.2, 0.4, 0.6, 0.8, 0.9-quantile of the p-values**)**. We show by additional simulations (Additional file 2: Figure S20, simulation details in the legend) that this test is very powerful and can detect a very moderate deviation from the assumption.  Therefore, in practice, we could apply both types of omnibus test simultaneously, and select covariates that are significant in the original test (i.e., overall dependency between p-value and covariate) and not significant in the suggested variant (i.e., the dependency is not due to violation of the independence assumption under the null).”

2.	I think that the results of Figures S9-S10 show one of the most useful results of the manuscript (the utility of the omnibus test in achieving performance gain with various methods). It also leads to questions about how to use these results to better interpret the results in Figure 3. It would be interesting to see a subset version of Figure three, which only gives results for a given dataset/covariate combination if the omnibus test passed a given threshold (missing otherwise). Or just gives the results for the most significant covariate per dataset (collapsed into 1 column).

**Answer and response:** *Thanks for this suggestion. We now give the results for the most significant covariate per dataset in Additional file 2: Figure S10 for more clarity.*

3.	The analyses using a p-value from a related disease/phenotype as a covariate (Figure 6): if the sets of p-values are too similar, the independence under the null assumption has potential to be violated. Has this been checked in this case?

**Answer and response:** *Thanks for the concern.  We now presented the stratified histogram (Additional file 2: Figure S18) to check the assumption of independence under the null.  We could see the p-value distribution is quite uniform in the large p-value region across strata, indicating the assumption is reasonable. We also conducted the variant of the omnibus test for detecting the assumption violation and achieved a p-value of 0.12, indicating there is no strong evidence of violation.*

4.	A note on possible interpretation of the reason AdaPT suffered from a great power loss when covariate is uninformative: Please see the section "Including an uninformative covariate is not harmful" in [1]. It seems that there is a simple remedy that can be invoked by the user when applying the AdaPT method, but the option is not made default nor mentioned in AdaPT's documentation despite being suggested by the authors of [1], which states that the AdaPT authors "responded positively to the recommendation of documenting this procedure in future releases of the package." A quick check reveals that this has not been done as of December 2019, and although I don't think it should not be the responsibility of the authors of this manuscript under review to implement this extra procedure on behalf of the AdaPT developers, it would be prudent to mention this issue since the findings are consistent with [1].

**Answer and response:** *Thanks for raising this important point. We now mentioned the issue in the discussion, which we also copied below:*

“However, when the signal became sparser and the covariate less informative, AdaPT suffered from a great power loss, which was consistent with the observation in [[45](https://docs.google.com/document/d/1L7tcxoSiMXCFDsCbk8JXrOH5rh6xwQjk/edit#heading=h.3dy6vkm)]. Although some remedy could be possibly invoked to compensate the power loss as suggested in [[45](https://docs.google.com/document/d/1L7tcxoSiMXCFDsCbk8JXrOH5rh6xwQjk/edit#heading=h.3dy6vkm)], no implementation has been available in the AdaPT package as of January 2020.”

    Minor comments:

    1.	Figure 1A: it is confusing to have the y-axis labeled as both Type I error and Power. Although it is clarified in the legend, I think interpretation of the figure would be easier if it was broken into two (one for the first column / Type I error and another for the second and third / power).

**Answer and response:** *Thanks. We have modified the figure.*

2.	Figure S6: are these results averaged over all 61 datasets? This should be clarified in legend.

**Answer and response:** *Yes, we have now made it clear in the legend.*

3.	The interpretation of the results of Figure 3 in the text does not point out how visually the CAMT method seems to perform the best. Indeed, there are a few cases where the performance is worse than ST, but this is to a lesser degree than any of the other methods.

**Answer and response:** *Thanks for pointing this out. The suggested new heatmap using the most informative covariate (Additional file 2: Figure S10) now visually shows CAMT/IHW are overall the best, although there is still power loss for several cases with very sparse signals.*

    References:

    [1] Korthauer K, Kimes PK, Duvallet C, Reyes A, Subramanian A, Teng M, Shukla C, Alm EJ, Hicks SC. A practical guide to methods controlling false discoveries in computational biology. Genome biology. 2019 Dec;20(1):118.

**Second round of review**

**Reviewer 1**

All my comments have been addressed.

**Reviewer 2**

The authors have satisfactorily addressed my concerns.
